# Implementation of Lost & Found, An Intervention to Reengage Patients Out of HIV Care: A Convergent Explanatory Sequential Mixed-Methods Analysis

**DOI:** 10.1007/s10461-022-03888-y

**Published:** 2022-10-22

**Authors:** Blake Linthwaite, Nadine Kronfli, David Lessard, Kim Engler, Luciana Ruppenthal, Emilie Bourbonnière, Nancy Obas, Melodie Brown, Bertrand Lebouché, Joseph Cox, the CVIS clinical team

**Affiliations:** 1 Research Institute of the McGill University Health Centre (RI-MUHC), 2155 Guy Street, 5th Floor, Montreal, QC H3H 2R9 Canada; 2 Department of Medicine, Division of Infectious Diseases and Chronic Viral Illness Service, McGill University, Montreal, QC Canada; 3 Department of Epidemiology, Biostatistics, and Occupational Health, Faculty of Medicine, McGill University, Purvis Hall, 1020 Pine Avenue West, Montreal, QC H3A 1A2 Canada; 4 Department of Family Medicine, Faculty of Medicine, McGill University, 5858 Chemin de la Côte des Neiges, Montreal, QC H3S 1Z1 Canada

**Keywords:** Implementation science, HIV care continuum, Lost to follow-up, Reengagement, Mixed-methods

## Abstract

Being out of HIV care (OOC) is associated with increased morbidity and mortality. We assessed implementation of Lost & Found, a clinic-based intervention to reengage OOC patients. OOC patients were identified using a nurse-validated, real-time OOC list within the electronic medical records (EMR) system. Nurses called OOC patients. Implementation occurred at the McGill University Health Centre from April 2018 to 2019. Results from questionnaires to nurses showed elevated scores for implementation outcomes throughout, but with lower, more variable scores during pre-implementation to month 3 [e.g., adoption subscales (scale: 1–5): range from pre-implementation to month 3, 3.7–4.9; thereafter, 4.2–4.9]. Qualitative results from focus groups with nurses were consistent with observed quantitative trends. Barriers concerning the EMR and nursing staff shortages explained reductions in fidelity. Strategies for overcoming barriers to implementation were crucial in early months of implementation. Intervention compatibility, information systems support, as well as nurses’ team processes, knowledge, and skills facilitated implementation.

**Supplementary Information:**

The online version contains supplementary material available at 10.1007/s10461-022-03888-y.

## Introduction

Identifying and reengaging out of HIV care (OOC) patients is important for optimizing engagement along the HIV care continuum [1, 2]. OOC patients experience greater morbidity and mortality [3–5], and increased risk of HIV transmission due to unsuppressed viral loads [6, 7]. Identifying and reengaging OOC patients requires consistent updates to patient data, including changes in living situation, HIV care, and contact information, which are often difficult to maintain [8]. Moreover, imprecise OOC definitions erroneously identify patients with valid reasons for not presenting to clinic, such as having changed care providers, moved away, or died, resulting in unnecessary efforts to reengage patients who are not actually OOC [8–13]. Methods to overcome these challenges include using large multi-jurisdictional datasets or data-sharing agreements, but these may be impeded by legal, financial, or infrastructural barriers [14–17]. Adapting existing, evidence-informed, clinic-based approaches for patient reengagement offers a viable alternative [18, 19].

Clinic-based approaches show promise for reengaging OOC patients [18, 19]. In addition to promoting patient reengagement, such efforts improve accuracy of patient care statuses and enhance overall data quality in clinical databases [8, 18–20]. However, beyond improvements in data quality, no studies have reported metrics to guide implementation of clinic-based reengagement interventions, nor have any been designed with an explicit focus on implementation [17]. Implementation science methods could help identify and mitigate challenges inherent to adopting such reengagement interventions [21].

Lost & Found is a clinic-based intervention for reengaging OOC patients, created and implemented at the Chronic Viral Illness Service of the McGill University Health Centre (CVIS-MUHC) [22, 23]. Lost & Found was designed to be adapted to varying contexts, and has successfully improved OOC patient reengagement at the CVIS-MUHC [23]. Insights gained from its implementation could guide efforts to adapt similar interventions elsewhere. We evaluated implementation of Lost & Found through mixed methods, describing quantitative trends in implementation outcomes and using qualitative data on implementation barriers and facilitators to provide additional context [24].

## Methods

### Lost & Found

**Fig. 1:**
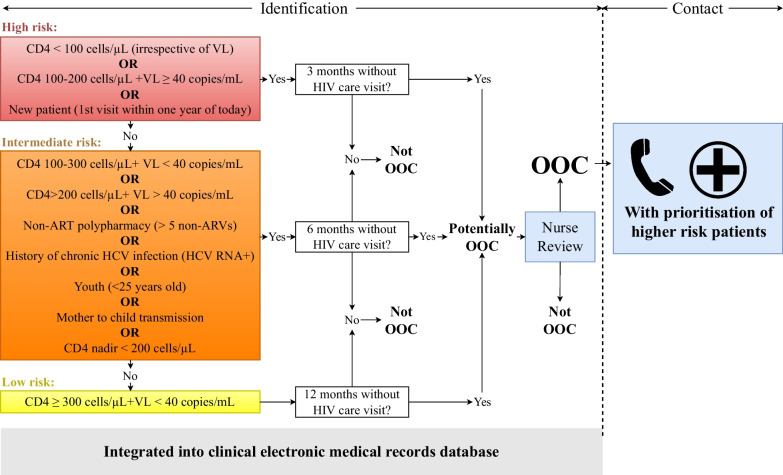
Schematic of the full Lost & Found intervention

**Fig. 2:**
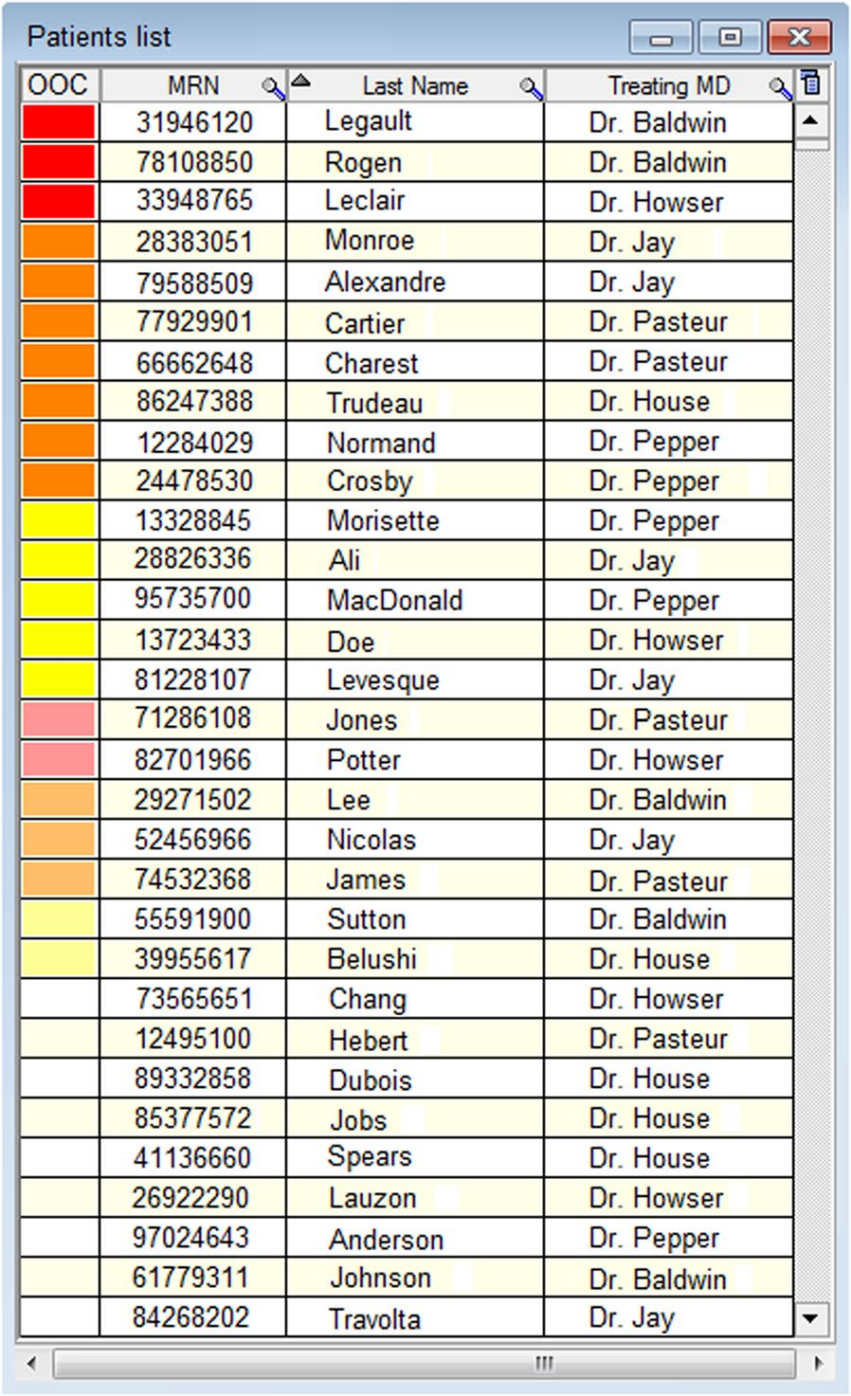
Out-of-care list built into the clinical electronic medical record database. *Note* Only fictional patient data are presented in this figure

**Fig. 3:**
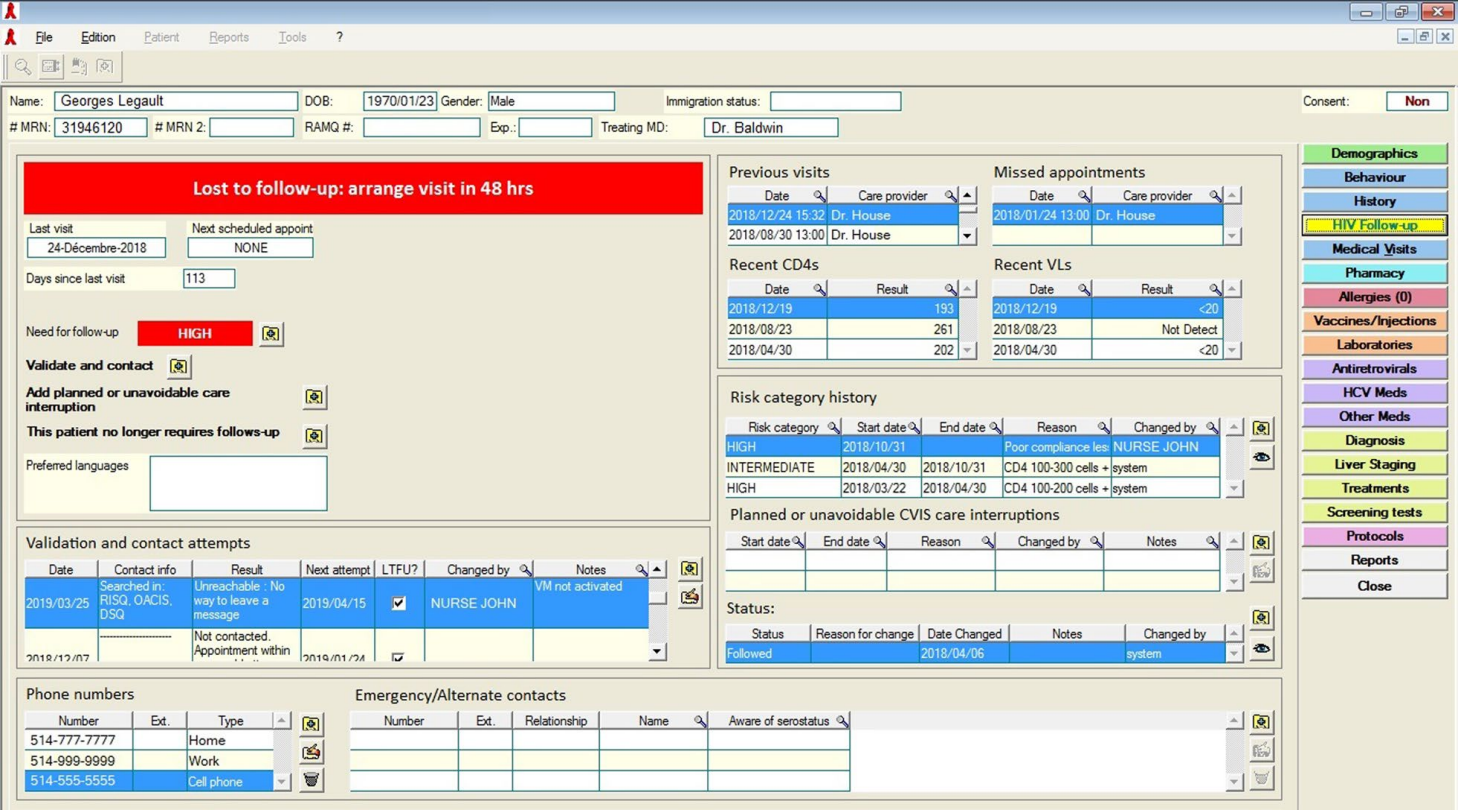
HIV care module built into the clinical electronic medical record database. *Note* Only fictional patient data are presented in this figure

Lost & Found consists of two core, evidence-based elements: identifying and contacting OOC patients (Fig. 1) [10, 18, 19, 22, 25, 26]. To identify OOC patients, we integrated an OOC risk prediction tool (OOC-RPT) into a pre-existing electronic medical records (EMR) database. The OOC-RPT uses clinical information (e.g., HIV-related lab results and demographic information) to automatically classify patients into high, intermediate, or low risk of negative HIV-related health outcomes and, based on this classification, as “potentially OOC” after 3, 6, or 12 months from their last HIV care visit, respectively [27, 28]. Specific criteria used to determine risk levels are detailed in Fig. 1. Potential OOC patients are organized into an “OOC list” to be reviewed by nurses who validate individual OOC statuses, based on their knowledge of a given patient’s sociodemographic, psychosocial, clinical, or other factors (Fig. 2). Nurses contact patients confirmed as OOC and document reengagement efforts in an HIV follow-up module we created in the database (Fig. 3). Nurses received training in motivational communication to facilitate patient reengagement.

Lost & Found was implemented at the CVIS-MUHC in Montréal, Canada over a 12-month period beginning in April 2018 [22]. A protocol outlining complete details of the intervention, its development, implementation strategies, and implementation frameworks is published elsewhere [22]. However, relevant information for this analysis is provided herein. We selected three implementation strategies to encourage uptake and sustainability of Lost & Found: (i) promote adaptability, wherein we differentiated *core elements*, critical to the intervention’s intent and effectiveness, from *peripheral components*, which can be adapted or adjusted [21, 29, 30]; (ii) planning, engaging, executing, evaluating, and reflecting (PEEER) cycles, or ‘cyclical tests of change,’ to adapt peripheral components of the intervention [21, 30]; and (iii) internal facilitation, whereby a study coordinator worked with stakeholders (nurses, primary investigators, database manager and developer) to identify and mitigate barriers to implementation [29, 30].

### Study Design

**Fig. 4:**
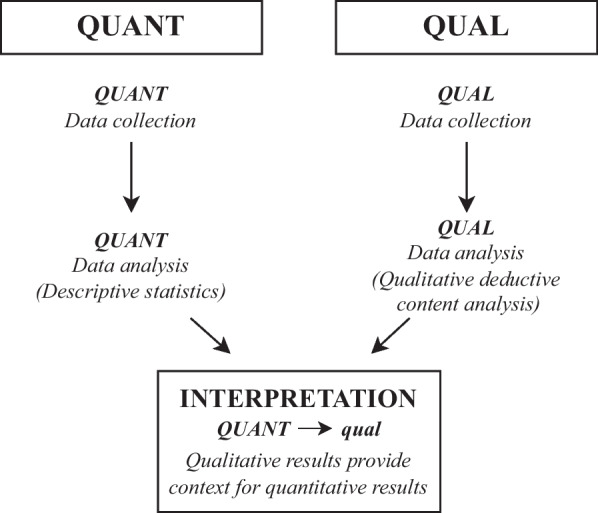
Convergent explanatory sequential mixed-methods design

This mixed-methods assessment of Lost & Found was conducted as part of a type II implementation-effectiveness hybrid study, where intervention effectiveness and implementation were given equal consideration [22, 31]. A completed Standards for Reporting Implementation Studies (StaRI) checklist is provided in Supplementary material 1 [32]. In a prior evaluation of the intervention’s effectiveness, we demonstrated that OOC patients were more likely to reengage and to do so earlier in the context of Lost & Found [23]. In the current assessment, we used a convergent explanatory sequential mixed-methods design to evaluate its implementation (Fig. 4) [22, 33, 34]. Qualitative and quantitative data were collected in parallel, then qualitative data were analyzed and used to contextualize quantitative findings.

### Data Collection and Analysis

Study participants included all nurses working at the CVIS-MUHC (n = 5), those responsible for administering the Lost & Found intervention. Quantitative and qualitative data collection at each time point are described in Table 1. Data collection tools are provided in Supplementary material 2 and details regarding the number of focus group discussions and related participation by nurses are provided in Supplementary material 3.

**Table 1:** Data collection schedule

	Pre-implementation	Week 1	Week 2	Week 3	Week 4	Month 2	Month 3	Month 6	Month 12
Feasibility	a, c				a, c	a, c	a, c	a, c	a, c
Acceptability	a, c				a, c	a, c	a, c	a, c	a, c
Adoption	a, c				a, c	a, c	a, c	a, c	a, c
Fidelity	b, c	b, d	b, d	b, d	b, c, d	b, c, d	b, c, d	b, c, d	b, c, d
Logbook*	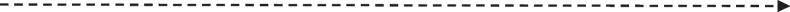								

(a) 36-item questionnaire; (b) fidelity checklist; (c) focus group; and, (d) EMRs**

*Feasibility* is the extent to which a new treatment or innovation can be successfully used or carried out within a given agency or setting;

*Acceptability* is the perception among implementation stakeholders that a given treatment, service, practice, or innovation is agreeable, palatable, or satisfactory;

*Adoption* is the intention, initial decision, or action to try or employ an innovation or evidence-based practice; and,

*Fidelity* is the degree to which an intervention was implemented as it was prescribed in the original protocol or as it was intended by the program developers [35]

^*^The study coordinator’s logbook contains information about pertinent events or conversations between study stakeholders that occurred throughout the study

**Patient-level data from EMRs is collected monthly from months 1 to 12

#### Quantitative Data

Four implementation outcomes were studied: (i) *feasibility*, (ii) *acceptability*, (iii) *adoption*, and (iv) *fidelity* [35]. A thirty-six item questionnaire (i–iii) and fidelity checklist (iv) were administered to nurses at pre-implementation (1 month before implementation) and months 1, 2, 3, 6, and 12. Scales for feasibility and acceptability were inspired by the feasibility of implementation measure (FIM) and acceptability of implementation measure (AIM), respectively [36]. Adoption was measured using scales from the Service Provider Adopter and Innovation Characteristics Questionnaire (SPAICQ) [37, 38]. Content validity, discriminant content validity, reliability, structural validity, structural invariance, known-groups validity, and responsiveness to change have been demonstrated for FIM and AIM, but predictive validity assessments are ongoing [36]. Overall, the SPAICQ rates as excellent for norms and good for usability [39]. Predictive validity is considered good for the SPAICQ adopter characteristics subscale, but minimal for the innovation characteristics subscale [39].

Feasibility and acceptability were measured separately for Lost & Found’s core elements (identifying OOC patients, contacting patients), while adoption was measured for the full intervention. Feasibility, acceptability, and adoption were captured via 5-item agreement scales and scored from one to five, where a score of one indicates complete disagreement, and a score of five, complete agreement. We created and administered a short checklist to assess fidelity to intervention components that could not be collected through EMRs (e.g. adherence to motivational communication techniques). These were scored on scales from zero to one, where a score of zero indicated either an answer of “no” to using the intervention component or “never” using it as intended, while a score of one indicated an answer of “yes” to using the intervention component or using it as intended “most of the time”. Scores are typically presented as the mean for all nurses at a given time point. When discussing scores across time points, we took the median and inter-quartile range (IQR).

Additional patient-level data from the implementation phase (months 1–12) were extracted from EMRs to further inform nurses’ fidelity to intervention components. Extracted data include the number of individuals identified as potentially OOC and, among those, the number validated by nurses, confirmed OOC, and who received contact attempts. Only patients with clinic visits within 5 years of the start of implementation and during implementation were included. We present monthly totals for each patient-level fidelity outcome.

#### Qualitative Data

*Focus Groups* One-hour audio-recorded focus group discussions were held with nurses in pre-implementation and months 1, 2, 3, 6, and 12, led by DL. Discussions took place in English and were guided by a semi-structured interview schedule. The study coordinator (BL^1^) was in attendance to learn about and, following the focus group discussions, address barriers to implementation mentioned throughout. BL^1^ and DL independently coded verbatim transcriptions of recordings in NVivo-12 using deductive content analysis [40, 41]. Transcripts were reviewed and coded for correspondence with items in the Tailored Implementation for Chronic Diseases (TICD) checklist, a comprehensive list of 57 implementation determinants (i.e. barriers and facilitators) [42]. Discrepancies were resolved by consensus.

*Logbook* Information about important events or conversations between stakeholders were recorded in a logbook by the study coordinator and used to further contextualize focus group results.

#### Mixed Methods

**Fig. 5:**
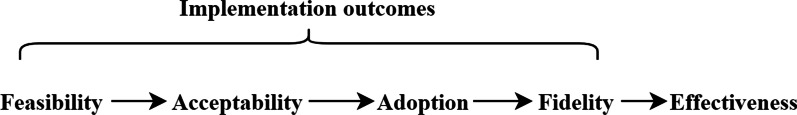
Simplified causal chain for study outcomes

To triangulate quantitative and qualitative data, we developed a convergence coding matrix to map TICD determinants onto implementation outcomes (Supplementary material 4) [42]. This process was guided by a simplified version of a causal chain we developed to understand the relationship between implementation outcomes and overall effectiveness (Fig. 5) [22, 35, 37, 43, 44].

The causal chain describes how each implementation outcome acts as an intermediate between upstream implementation outcomes and intervention effectiveness. Upstream implementation outcomes such as feasibility and acceptability are fundamental to adoption, fidelity, and ultimately effectiveness [22]. Determinants directly impacting an upstream implementation outcome indirectly impact downstream outcomes, but do not impact upstream ones. For example, if implementation stakeholders do not accept the intervention, it could still be inherently feasible to implement, but the lack of acceptability will prevent them from adopting it, ultimately rendering it ineffective.

Mixed-methods analyses were first performed by BL^1^, who explored trends in implementation outcomes using focus groups results, further contextualized by the logbook. To quantify the importance of a theme, we examined coverage, defined as the proportion of words coded as aligning with a determinant, a useful proxy when there is a large number of potential codes, as was the case in this study due to the large number of determinants [41, 45]. The top 10 barriers and facilitators discussed across focus groups, as determined by their coverage, were prioritized to explain quantitative trends, supported by the convergence coding matrix. Notably, determinants could act as both barriers and facilitators. Transcripts were reviewed again, and other potentially important events not identified in the 10 barriers and facilitators were considered. BL^1^ provided an inclusive account of potentially important determinants and events, after which they were collectively reviewed by BL^1^, DL, and JC to determine which best explained trends in implementation. Disagreements were resolved by consensus. TICD determinants are italicized throughout the text.

## Results

The following sections describe patterns for each implementation outcome. These results are triangulated and explained by findings from qualitative content analyses. Supplementary material 5 summarizes how each determinant impacted implementation outcomes during implementation.

### Feasibility

**Fig. 6:**
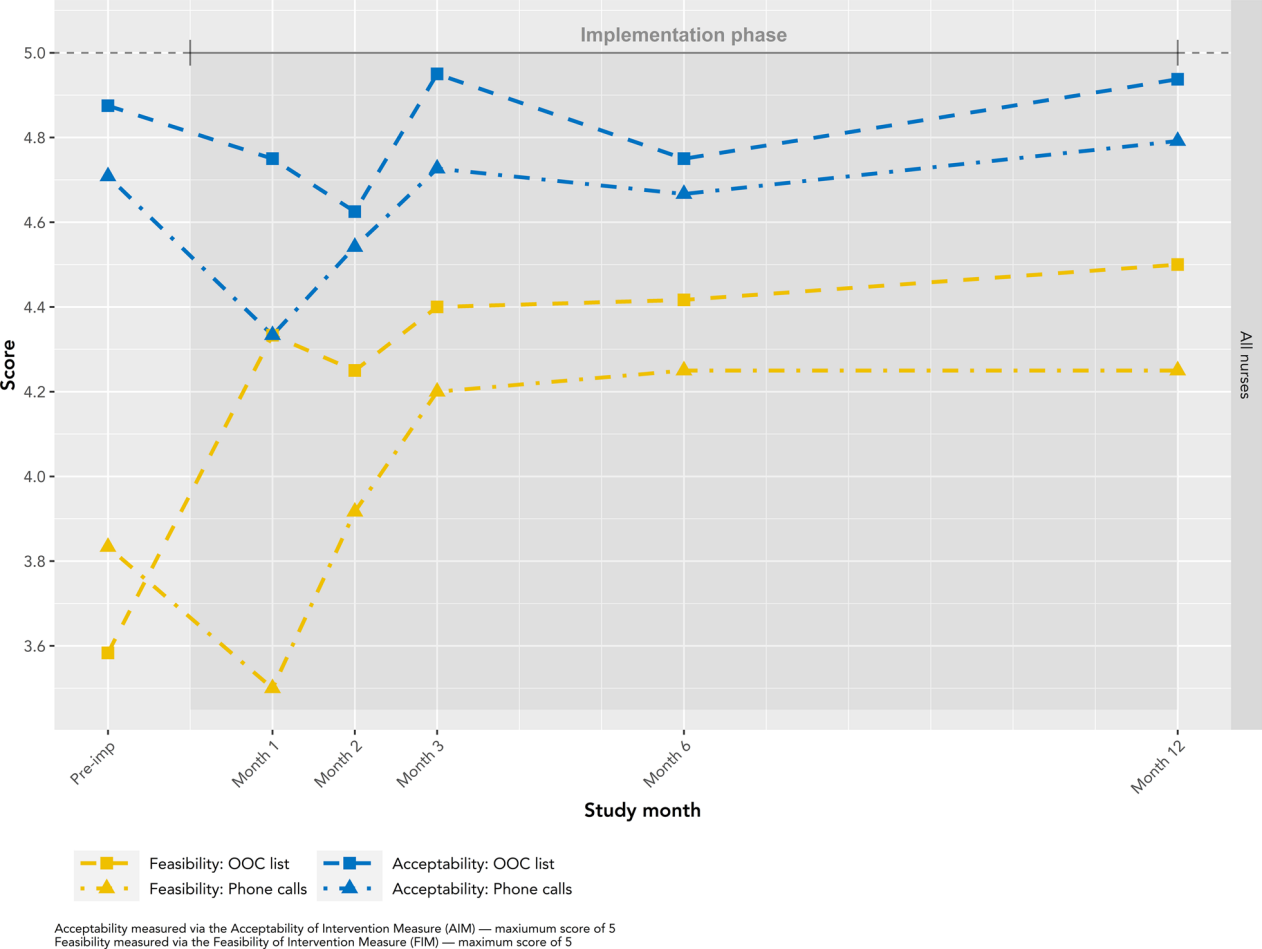
Feasibility and acceptability of the phone calls and OOC list, all nurses by study month

For each core element of the intervention (identifying OOC patients, contacting patients), nurses’ feasibility scores were lower and more variable in early study months (pre-implementation to month 3), followed by higher scores in later months (months 3–12) (Fig. 6). In pre-implementation, nurses perceived managing the OOC list to be less feasible (score of 3.6), but this outcome improved throughout implementation (median 4.4; IQR: 4.3–4.4), with a slight decline in month 2 to a score of 4.2. Conversely, in pre-implementation, nurses perceived phone calls as more feasible (score of 3.8) than managing the OOC list but reported lower feasibility in month 1 (score of 3.5). In month 3, feasibility of phone calls plateaued at 4.2, just below perceived feasibility of the OOC list.

Determinants directly impacting feasibility dominated focus group discussions. These included *nature of the behaviour, information systems, assistance for organizational changes, compatibility, availability of necessary resources, and patient behaviours, needs, and preferences.*

For identification of OOC patients, nurses recognized the inherent characteristics of this task (i.e. *nature of the behaviour*) as an important barrier during the pre-implementation focus group. They knew they would have to confirm statuses of patients on the OOC list on a case-by-case basis, many of whom would no longer be in care due to changing clinics or relocating to another jurisdiction.

Assuming in the beginning, there’ll be a long list of patients, we probably won’t be able to get through them all right away.

-Nurse 1, pre-implementation

This concern likely contributed to low feasibility scores for managing the OOC list in pre-implementation. To make the list more manageable, following consultation with nurses and primary investigators, at the start of implementation the study coordinator removed patients without a visit in five years. Despite this, nurses had to assess the OOC status of 862 patients remaining on the list. The coordinator’s involvement in this matter, and support from clinical *leadership* who made temporary nurses available to conduct other day-to-day tasks, provided the necessary resources to facilitate “cleaning” the OOC list and implementation overall. Improvements to *information systems* (i.e., EMRs) also played an important role in implementation, including nurses’ views of the intervention as efficient, helping make reengagement easier and more sustainable.

We’ll be able to [identify patients] more systematically. We’ll be able to catch more that otherwise might fall through the cracks.

– Nurse 1, pre-implementation

Consequently, nurses’ views of the OOC list in month 1 are mostly positive, and feasibility scores increased after pre-implementation. However, perhaps due to the central role of the EMR database in Lost & Found implementation, *information systems*-related barriers were among the most frequently discussed, particularly in early implementation. For example, before the month 2 focus group, to improve functioning of the OOC list, the study coordinator changed how “HIV care visits” were defined such that laboratory measures without consultation with a member of the clinical team were no longer considered. This change resulted in a significant increase in the number of patients on the OOC list and likely contributed to the concomitant decline in feasibility scores. However, nurses acknowledged that many barriers, especially for *information systems*, were addressed by notifying the study coordinator, who provided *assistance for organizational changes* related to any aspect of the project:

[The study coordinator] is responsive and answers quickly. If there’s a solution, there’s usually something that’s found pretty quickly.

-Nurse 1, Month 3

By collaborating with the primary investigators, database manager, and other clinical staff, the study coordinator ensured that essential software updates were installed when problems arose. Ten updates to the EMR database were installed in the first 4 months, compared to three in the remaining eight. Only one update was made after month 7, and it had no impact on monitoring or reengaging OOC patients. Timing of these changes is consistent with lower and more variable scores for feasibility in early versus later study months. Supplementary material 6 contains a full history of intervention-related changes to the database.

Once identified, nurses attempted to reengage OOC patients with a phone call. *Compatibility* of this part of the intervention was consistently recognized as a facilitator. Before Lost & Found, nurses made their own efforts to call patients perceived as potentially OOC, likely contributing to compatibility of the intervention and potentially explaining higher pre-implementation feasibility scores for phone calls compared to managing the OOC list. Nurses explained that, before the intervention, they had invested time into reengaging patients they perceived as OOC, but that Lost & Found helped them accomplish this task more effectively:

We were already doing phone calls before; it’s part of the job. It’ll just be easier to do than before.

-Nurse 2, Month 6

Nurses considered monitoring and reengaging OOC patients as central to their nursing responsibilities and consistent with the culture of the clinic, further contributing to *compatibility*.

*Availability of necessary resources* was a frequently discussed barrier. Limited nursing resources remained a recurring issue throughout implementation:

If one of us is on vacation there’s only three of us. Then we’re busy enough with just the clinic stuff that we won’t necessarily have time to do it.

-Nurse 3, Month 12

This barrier may have contributed to lower feasibility scores throughout, including the decline from pre-implementation to month 1, when one nurse was absent.

Nurses also highlighted *patient behaviours, needs, and preferences* as barriers. For instance, some patients would not present despite a planned appointment. Nurses acknowledged that the main barriers preventing reengagement stemmed from challenges patients faced in their day-to-day lives. For example, until month 9, nurses were selecting “Other” over “Unwilling to engage in care” as the outcome of their reengagement efforts when patients refused reengagement, because they felt that “unwilling” was a misrepresentation of barriers faced by patients in accessing health care:

If they live far, if they have to ask to have time off, [… if they have to] get home in time to pick up kids from school, it constrains people into a small window that they can come to the clinic.

-Nurse 1, Month 6

Some of the barriers mentioned included non-HIV-related health issues, family and work responsibilities, as well as transportation issues.

Nurses also frequently expressed concern about potentially making patients feel confronted or guilty about their absences from care. It was feared this may discourage patients from reengaging altogether:

I’m trying not to confront them. I’m trying to be positive.

-Nurse 4, Month 2

Similar patient-related challenges persisted throughout the study, likely contributing to lower feasibility scores for phone calls compared to managing the OOC list.

### Acceptability

Acceptability scores were high throughout the entire study, with patterns in acceptability of the OOC list and phone calls similar to those observed for feasibility (Fig. 6). A deviation from this pattern was observed in pre-implementation when the average acceptability score for managing the OOC list (4.88) was higher than for phone calls (4.71), while the average feasibility score for the OOC list (3.58) was lower than for phone calls (3.83). After pre-implementation, acceptability scores for the OOC list declined to their lowest in month 2 (average 4.62), then increased to their highest in month 3 (average 4.95). This decline mirrors the concomitant fall in feasibility scores. Thereafter, scores remained above 4.75 until study completion. Similar to feasibility scores, acceptability scores for phone calls decreased from pre-implementation (average 4.71) to month 1 (average 4.33) before plateauing near 4.7 in month 3.

*Observability* of the intervention was the most frequently reported facilitator directly affecting acceptability. Nurses consistently highlighted benefits of seeing the OOC list in real-time, including the categorization of patients into different risk categories, while simultaneously acknowledging limitations in functionality of the OOC list and related *information systems* in early implementation.

Little nicks in [the database] but generally it’s good. It’s kind of exciting to see when it works. When it pops up a name at the top and you say: ‘Oh, yes, that’s definitely an [out-of-care] patient’

-Nurse 1, Month 1

As an added facilitator of acceptability, nurses were among the primary *sources of the recommendation*, meaning they were central in developing Lost & Found, and relied upon previous reengagement efforts to do so. Nurses were frequently consulted in pre-implementation to ensure that proposed adaptations met their needs. Across focus groups, they made statements such as the following:

I’m looking forward to it. We’ve been asking for something. We’re always doing it anyways but hopefully with the tool, it’ll be more systematic.

-Nurse 1, pre-implementation

The central role of nurses likely contributed to high acceptability scores throughout the study and may explain why perceived acceptability scores consistently exceeded perceived feasibility across focus groups.

Nurses’ experience with patient reengagement, and their role in Lost & Found development, may also explain the discordance between pre-implementation acceptability and feasibility scores for the OOC list. Specifically, nurses may have been happy with its purpose (acceptability), but skeptical about how it would actually work (feasibility). The concordance in acceptability and feasibility trends for the OOC list during implementation suggests their pre-implementation concerns were adequately addressed.

### Adoption

**Fig. 7:**
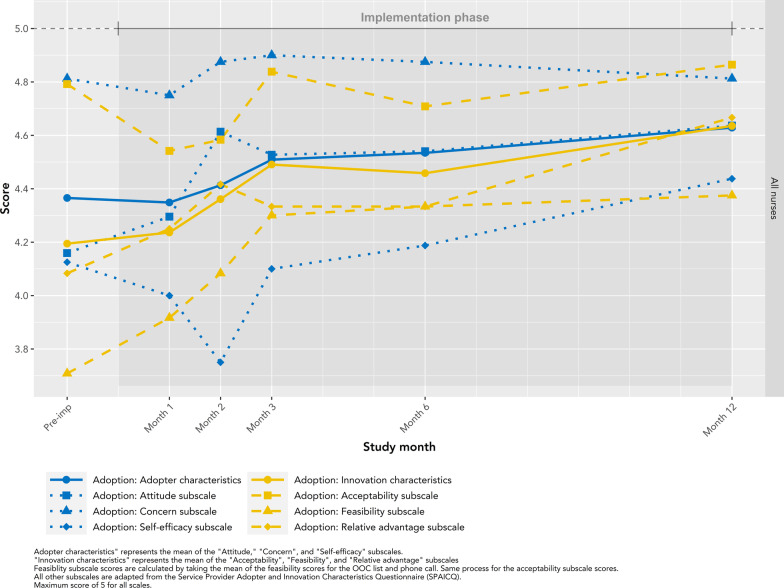
Adoption subscales for Lost & Found, all nurses by study month

High levels of adoption were observed throughout the study (median score across subscales 4.4; IQR: 4.2–4.7), with more variability in pre-implementation to month 3 (range of scores from pre-implementation to month 3, 3.7–4.9; thereafter, 4.2–4.9) (Fig. 7). For all adoption subscales, average scores were higher in month 12 (median 4.7; IQR: 4.5–4.8) than in pre-implementation or month 1 (median 4.2; IQR: 4.1–4.6). We observed three general patterns for adoption subscales: (i) high “concern for OOC patients” scores were seen throughout the entire study (median: 4.8; IQR: 4.8–4.9); (ii) nurses’ self-efficacy and overall acceptability of Lost & Found dropped slightly in months 1 and 2 from pre-implementation (mean decrease of 0.24), then increased in month 3 (mean increase of 0.25); (iii) attitudes toward Lost & Found, its relative advantage compared to standard practice, and overall feasibility increased throughout the study from their lowest levels in pre-implementation to their highest in month 12 (median increase 0.58; IQR: 0.53–0.62), with some minor fluctuations throughout. The two aggregate adoption scales, adopter characteristics (an average of attitude, concern, and self-efficacy) and innovation characteristics (acceptability, feasibility, and relative advantage), each followed the third pattern (median increase of 0.35 from pre-implementation; IQR: 0.31–0.40). Consistent with the causal chain of implementation outcomes, patterns in adoption scores were consistent with those observed for feasibility and acceptability—slight declines in months 1 and 2, followed by either an increasing trend or consistently high scores.

These trends seem to be largely explained by determinants impacting upstream implementation outcomes. For example, declines in self-efficacy in the first 2 months of implementation were likely related to feasibility, more specifically, nurses’ concerns about their ability to schedule patients with their doctors, related to the *availability of necessary resources* and *referral process* determinants:

Summer is coming so it’s been harder now because, for most doctors, the next availability is in [two months]. The longer it takes for an appointment, the less chance we have for the patient to show.

-Nurse 2, Month 2

However, two determinants, *domain knowledge* and *skills needed to adhere*, were among the most frequently discussed facilitators to adoption. Here, “domain knowledge” refers to nurses’ knowledge about OOC patients and HIV in general. Nurses at the CVIS-MUHC specialize in HIV care, meaning these two determinants likely exerted strong positive effects on adoption throughout. For example, patients could be prematurely identified by the EMR database when their upcoming appointments were just outside of the risk-category-specific window for amount of time since their previous clinical visit; a patient in the high-risk category with an upcoming appointment in 3 months and 1 week after their last clinical visit would be identified by the database as of the 3-month mark until they successfully attended their appointment or were removed from the OOC list. Nurses had strong existing relationships with many OOC patients, allowing them to differentiate truly OOC patients from those who were erroneously or prematurely identified by the EMR database, which they anticipated would facilitate adoption of the intervention:

There [are] so many patients that we could quickly remove just by looking at the list: ‘Oh, he’s at another clinic, he’s there, he’s there.’

-Nurse 2, pre-implementation

As highlighted when discussing feasibility, nurses’ *domain knowledge* also meant they understood many of the barriers faced by patients in remaining engaged in care, such as competing priorities and essential needs related to work or family responsibilities. This is reflected in their consistently high levels of concern for OOC patients. Their understanding of OOC patients likely contributed to their skills in reengaging patients over the phone, and ultimately their self-efficacy in encouraging patient reengagement:

[Patients] don’t know [nurse 2], but when she calls, she’s open and people feel a little more inclined to want to come back.

-Nurse 1, Month 6

### Fidelity

**Fig. 8:**
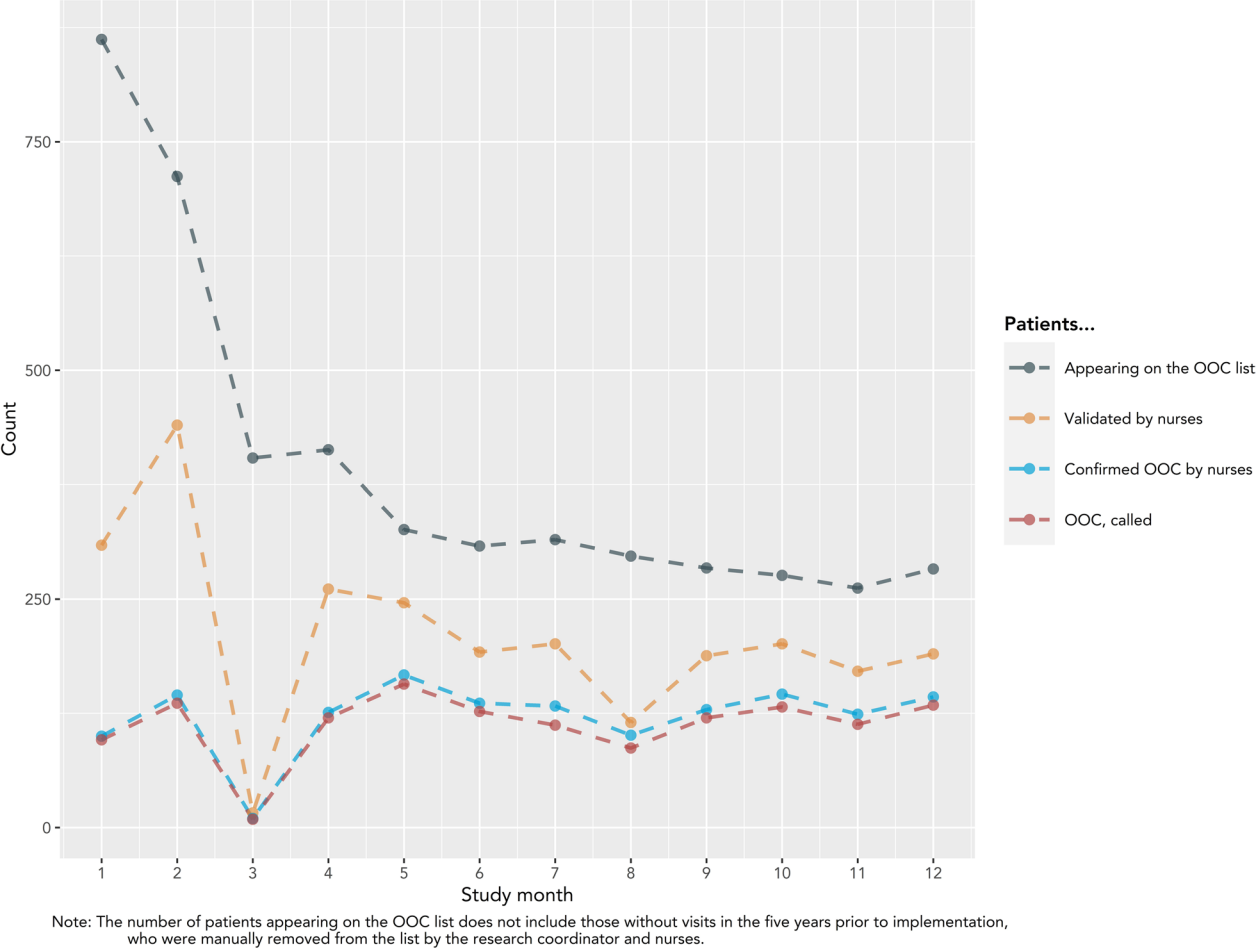
Fidelity outcomes from patient-level data in EMRs

**Fig. 9:**
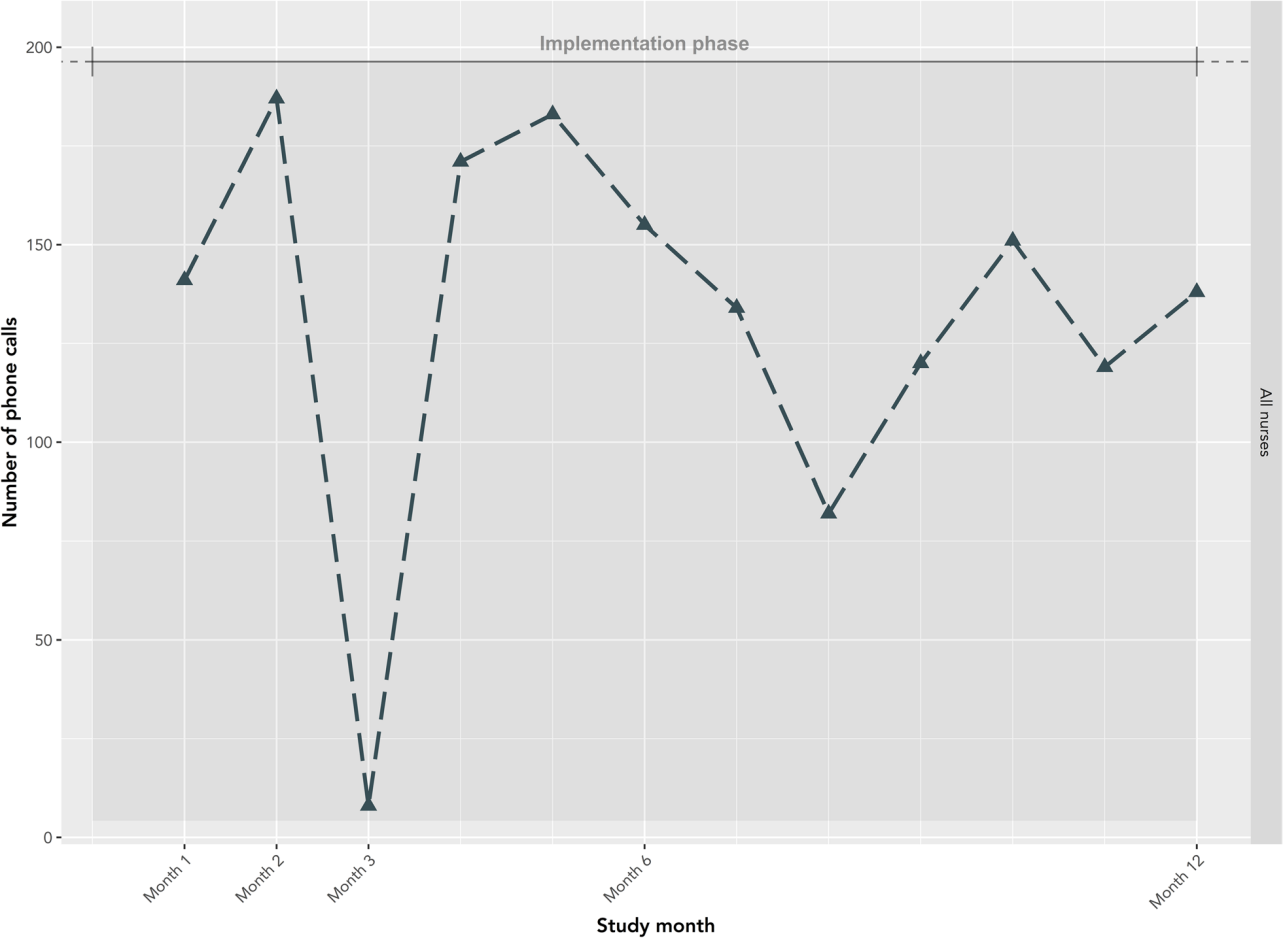
Number of phone calls made, all nurses by month

**Fig. 10:**
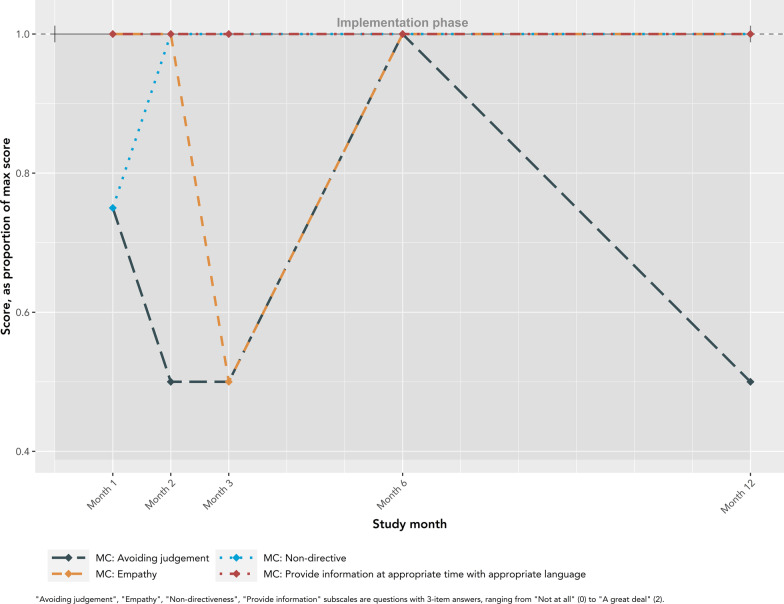
Fidelity to motivational communication principles, Nurse 2* by study month. *Nurse 2 made 92.1% (1464/1589) of the phone calls throughout the study

**Fig. 11:**
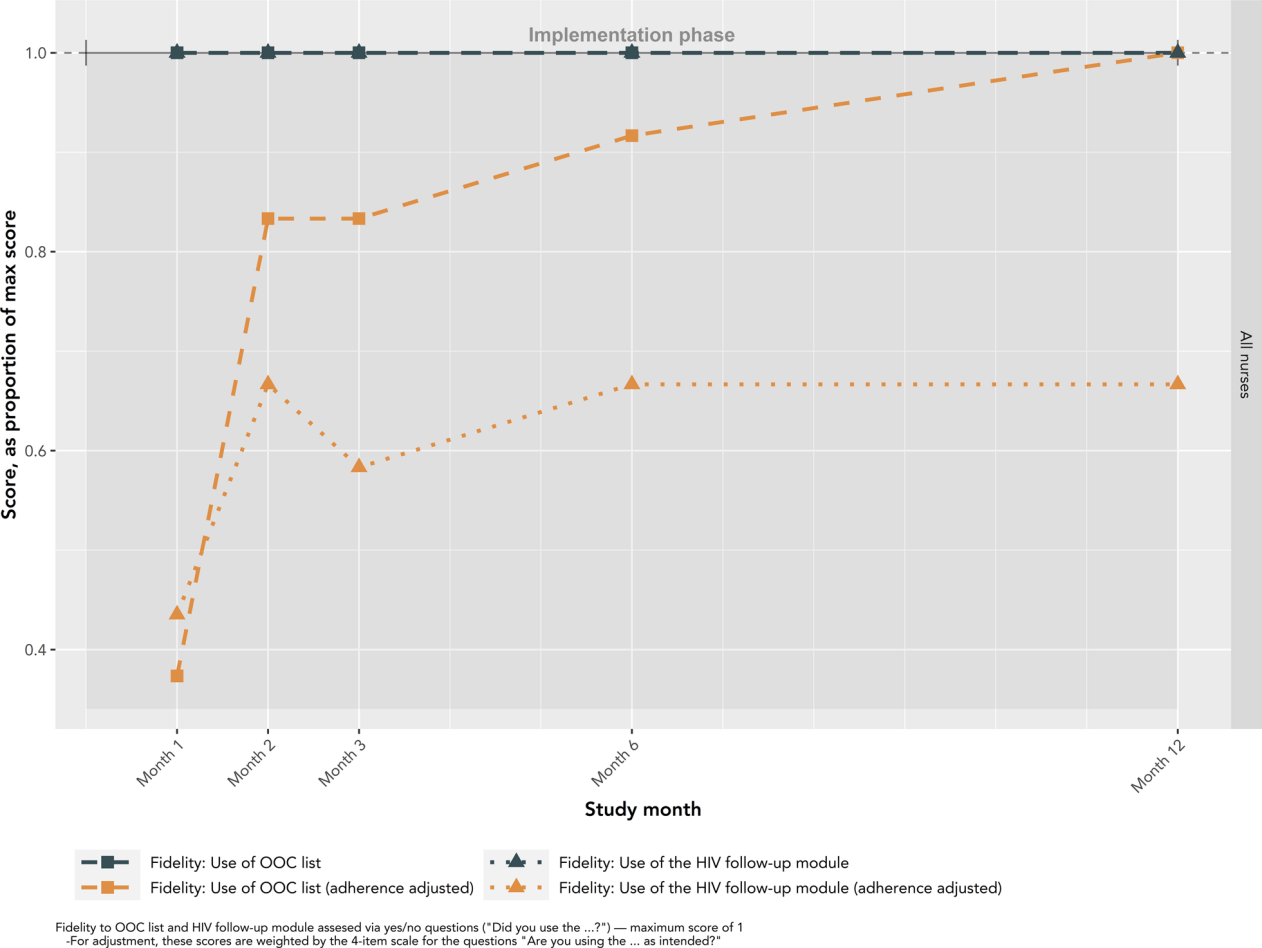
Use and adherence to the OOC list and HIV follow-up module, all nurses by study month

Fidelity outcomes from patient-level data from EMRs are presented in Fig. 8. The number of patients on the OOC list declined from its peak of 862 in month 1 to a plateau (median 290; IQR: 281–310) beginning in month 5. Similarly, the highest number of patients were validated by nurses in the first 5 months, with a peak of 440 in month 2, followed by a plateau starting at month 6 (median 190; IQR:180–196). There were noticeable declines in patients validated in months 3 and 8. The number of patients confirmed as OOC, and subsequently called by nurses, was relatively stable across the study, following a similar pattern to the number of patients validated by nurses. True OOC patients made up an increasing proportion of patients on the list over the study. In month 1, true OOC patients made up only 12% of patients, compared to an average of 47% in months 5 to 12. Similar patterns were observed when stratified by risk category (Supplementary material 3). The number of calls made by nurses each month are presented in Fig. 9. One nurse, nurse 2, made 92.1% (1464/1589) of the calls throughout the study. This nurse reported consistently high adherence to the general principles of motivational communication, particularly empathetic, non-directive, and informative communication (Fig. 10). The other four nurses completed a maximum of 41 and a median of 7.5 [IQR: 2.5–12.5] calls in any given month. Nurses completed the fewest calls in months 3 and 8, consistent with lower validation rates during these months. All nurses reported using the OOC list and HIV follow-up module (where reengagement efforts are documented) throughout the study (Fig. 11). Increasingly over the study period, nurses used the OOC list as intended, defined as prioritizing patients with the longest clinical absences in the highest risk categories. Nurses tended to report using the HIV follow-up module as intended “some of the time” (mean score 0.67), where “as intended” is defined as conducting all reengagement activities in the EMR database and not elsewhere.

Based on the qualitative analyses, nurses’ *knowledge of their own practice* was the most frequently occurring facilitator of fidelity. This allowed them to overcome inherent limitations to the EMR database and obtain patient information from other sources such as health registries. Similarly, nurses’ *team processes* facilitated fidelity to other aspects of Lost & Found. The five nurses emphasized a shared mandate, with individual, overlapping responsibilities:

We have different responsibilities. I think we manage to work it out amongst each other. There’s no clear division, we try to help each other.

- Nurse 4, month 12

Initial management and ‘clean-up’ of the OOC list were accomplished as a group, but nurse 2 remained primarily responsible for OOC patient reengagement throughout the study. This division of labour occurred naturally, with nurses insisting that no specific nurse be formally assigned to lead reengagement efforts. Notably, despite the disparity in phone calls made, across all implementation outcomes, scores are relatively consistent between nurses (Supplementary material 3).

As with adoption, observed trends in fidelity are largely explained by determinants impacting upstream implementation outcomes (feasibility, acceptability, and adoption). For example, there was a decrease in the number of patients validated, confirmed as OOC, and called (e.g. in months 1 and 3) when any nurse, especially nurse 2, was sick or on vacation, limiting the *availability of necessary resources*. In such instances, the remaining nurses would prioritize reengagement of high-risk patients, if any.

Similarly, *information systems*, an important determinant acting through feasibility, also impacted fidelity scores. For example, the increasing trend in use of the OOC list as intended is consistent with database improvements over the course of implementation, suggesting these changes facilitated fidelity. *Information systems* as a barrier also explains other trends in fidelity. For example, in month 8, two nurses were unable to access EMRs on their computers due to hospital server upgrades, ultimately leading to a concomitant decline in validated patients (Fig. 8). While nurses were able to access the software in subsequent months, access was inconsistent and not completely resolved until after implementation, when new computers were installed. Similarly, use of the HIV follow-up module as intended only “some of the time” (Fig. 11) likely reflects inherent limitations of data in the EMR database, forcing nurses to search elsewhere for patient information.

Finally, nurses’ focus on accommodating patients likely contributed to high levels of adherence to the general principles of motivational communication. Notably, they viewed these principles as part of their expertise as nurses, not necessarily as part of motivational communication.

## Discussion

In this mixed-methods study, we examined implementation of an evidence-informed clinical intervention to reengage people living with HIV into care. To our knowledge, this is the first study to employ an implementation science or mixed-methods approach to clinic-based HIV care reengagement [17]. We found initial months to be crucial to successful implementation, while later months were essential to improving the fit of the intervention for the clinic. This is consistent with Proctor’s proposed salience of implementation outcomes, whereby early implementation is considered a critical time period for measurement and adaptation [35]. These findings are also consistent with other studies finding improved clinical data as a major benefit of HIV patient reengagement or retention efforts [8, 20]. As nurses validated patients’ OOC statuses over time, automated portions of our OOC definition became more specific, easing the burden on nurses.

Our results demonstrate that implementation of Lost & Found was rooted in the three selected implementation strategies: promoting adaptability, internal facilitation, and PEEER cycles. Packaging the intervention into peripheral components and core elements allowed us to delineate which aspects could be adapted, and which should remain untouched, respectively. Any aspect of the intervention adapted to the clinic or changed throughout implementation represents a peripheral intervention component. This strategy was apparent across adaptations, including changes to the EMR database and OOC criteria. Internal facilitation and PEEER cycles also played important roles in adapting the intervention. During early implementation, the study coordinator worked with various implementation stakeholders to ensure that timely changes to peripheral components were implemented. Nurses acknowledged that internal facilitation was a useful resource in addressing barriers to implementation. These changes were possible because the study coordinator was given resources to implement necessary changes, including finances for a software developer and support from clinical leadership to engage with stakeholders in addressing unanticipated challenges. This flexibility allowed the study coordinator to document, resolve, and monitor issues in a manner consistent with informal PEEER cycles conducted throughout [21]. Notably, a study coordinator acted as our internal facilitator, but other clinics might consider assigning this role to another member of the clinical team. We chose a study coordinator as they had no competing clinical responsibilities, but this may have come at the expense of closer contact with nurses.

Some determinants, such as *information systems* and *availability of necessary resources,* appeared to be directly tied to events impacting implementation outcomes, as demonstrated by decreases in the number of patients validated by nurses in months 3 and 8*.* Reengagement interventions tend to require significant effort and resources, meaning *availability of necessary resources* is likely to be pertinent regardless of where Lost & Found is implemented [11, 16, 46]. The predominance of *information systems* as a determinant in our clinic was likely related to our strategy to overcome the resource-intensive nature of identifying OOC patients and documenting reengagement efforts. Specifically, our OOC definition placed technology at the center of our approach. This is exemplified by the decline in patients validated in month 8; problems with accessing or the software itself prevented or limited reengagement activities. While *information systems* would be a less important determinant in clinics adopting Lost & Found with an OOC definition less dependent on technology, a new set of challenges would arise that were mitigated using our approach. For example, nurses requested a more complex OOC definition with a low threshold for identification, to ensure a larger proportion would be captured. With our approach, we could integrate their request into the EMR and automate some of their work. A simpler and higher threshold definition, such as one considering only patients with 12-month absences as OOC, could be less dependent on technology and potentially reduce nurses’ workload, at the expense of depriving some OOC patients from receiving reengagement efforts. Clinics using a different OOC definition would have to find ways to overcome its limitations, including how it is operationalized and accepted by clinical staff. Conversely, for contacting patients, some clinics may opt for additional methods, including text messages or emails. These strategies have been employed in other reengagement studies and warrant further study [17].

Other determinants seemed to have exerted their effects before data collection. Multiple discussions were had between implementation stakeholders that may have played an important role in tailoring the intervention to the clinic. The first formal meeting between nurses and primary investigators occurred in March 2017, just over a year before the start of implementation and data collection. Determinants such as *compatibility* and *source of the intervention* were discussed frequently across focus groups, but likely exerted their effects on implementation outcomes in these early stages of Lost & Found conception and development, helping to adapt the intervention for eventual implementation. Similarly, other determinants rarely mentioned throughout focus groups, such as *quality of evidence supporting the recommendation,* may have been important, but not discussed because they were considered obvious and inherent to discussions that occurred before data collection. In September and October 2017, we completed modified versions of the worksheets from the TICD framework to select our implementation strategies as a function of potential barriers and facilitators to successful implementation [22, 42]. This process may have allowed us to avoid certain barriers and capitalize on facilitators present in our clinic, allowing us to establish a foundation for the intervention throughout implementation. Our high pre-implementation scores may partially reflect this work done a priori. For example, *observability* of the OOC list was prioritized early in Lost & Found development and was a frequently mentioned facilitator across focus groups. It may be important to achieve high implementation scores in pre-implementation to tolerate a decline in early implementation, when most adaptation occurs.

While results of this study could help guide implementation of Lost & Found in different contexts, there are some notable limitations. First, this study was conducted at a single site, a multidisciplinary clinic in a publicly-funded quaternary care hospital in Canada. Some of our findings may not be generalizable to clinics with fewer financial or human resources, perhaps facing barriers not discussed here. Second, our findings are drawn from a small sample of stakeholders, which could further limit generalizability of our findings. We attempted to overcome this by corroborating findings from multiple data types and sources, including questionnaires, focus groups, and the facilitator’s logbook. Third, the scales used to measure implementation outcomes (FIM, AIM, and SPAICQ) are relatively new, and evidence regarding their validity and reliability is still accumulating [36, 39]. Fourth, some changes initiated and implemented without involvement from the study coordinator, or not mentioned in focus groups by nurses, may not have been accounted for or documented in this analysis. These could include changes in the distribution of work between nurses or their approach to phone calls. For example, despite nurses having collectively requested a shared responsibility for Lost & Found tasks and preferring to not assign a primary “Lost & Found nurse”, over time, we observed that one nurse made the large majority of phone calls to OOC patients. Fifth, CVIS-MUHC nurses were highly motivated to reengage OOC patients, even prior to Lost & Found. This strong facilitator for implementation may limit the generalizability of our findings to clinics with less buy-in from implementation stakeholders. Finally, our overall approach to this assessment focused solely on service providers, recognizing their essential role in implementation-related adaptations in order to, ultimately, improve patient outcomes. This approach aligns with Proctor’s evaluation framework for implementation research, whereby implementation outcomes are fundamental to service outcomes (e.g. effectiveness), which themselves influence client outcomes (e.g., patient satisfaction) [35]. A forthcoming analysis of questionnaires administered to patients upon reengagement will explore reasons for OOC to help guide the clinical team in its ongoing efforts to reengage and retain patients.

## Conclusions

This study provides important insights into the implementation of Lost & Found, an evidence-informed clinic-based reengagement intervention for OOC patients. Early months of the intervention were crucial for successful implementation. Important determinants of implementation were identified that may influence future efforts to trial this intervention, including information systems, availability of necessary resources, compatibility of the intervention, and health professionals’ skills and knowledge. Implementation strategies were important for overcoming barriers to implementation. These findings could be leveraged to overcome challenges and determine priorities in scaling up Lost & Found for more robust effectiveness and implementation evaluations, including cost-effectiveness studies.

## Supplementary Information

Below is the link to the electronic supplementary material.

Supplementary file1 (DOCX 81 KB)

Supplementary file2 (DOCX 67 KB)

Supplementary file3 (DOCX 2885 KB)

Supplementary file4 (XLSX 64 KB)

Supplementary file5 (DOCX 20 KB)

Supplementary file6 (DOCX 1681 KB)

## Data Availability

The datasets generated and/or analysed during the current study are not publicly available due to the risk of participants being linked to their statements and the resulting impact on their employment. However, data can be made available from the corresponding author upon reasonable request.

## Abbreviations

HIV: Human immunodeficiency virus
OOC: Out of HIV care
CVIS-MUHC: Chronic Viral Illness Service of the McGill University Health Centre
OOC-RPT: OOC risk prediction tool
PEEER: Planning, engaging, executing, evaluating, and reflecting
FIM: Feasibility of implementation measure
AIM: Acceptability of implementation measure
SPAICQ: Service Provider Adopter and Innovation Characteristics Questionnaire
IQR: Inter-quartile range
TICD: Tailored Implementation for Chronic Diseases
