# Supplementary material for: Implementation of Lost & Found, An Intervention to Reengage Patients Out of HIV Care: A Convergent Explanatory Sequential Mixed-Methods Analysis
Source: AIDS Behav. 2022 Oct 22;27(5):1531–47. doi: 10.1007/s10461-022-03888-y (PMC10130100; doi:10.1007/s10461-022-03888-y)

Supplementary material 4: Additional graphs for implementation outcomes

This document provides additional graphs for implementation outcomes measured in the Lost & Found study, stratified by participant (nurses) or risk category, depending on the outcome.

**Table of Contents**

[Feasibility and acceptability 2](#_Toc98497736)

Figure A1: Feasibility and acceptability of the phone calls and OOC list, by nurse, by study month2

[Adoption 3](#_Toc98497737)

Figure A2: Adoption scales for Lost & Found, by nurse, by study month3

[Fidelity 4](#_Toc98497738)

Figure A3: Fidelity outcomes from patient-level data in EMRs, by risk category, by month4

Figure A4: Use and adherence to the OOC list and HIV follow-up module, by nurse, by study month5

# Feasibility and acceptability





# Adoption





# Fidelity


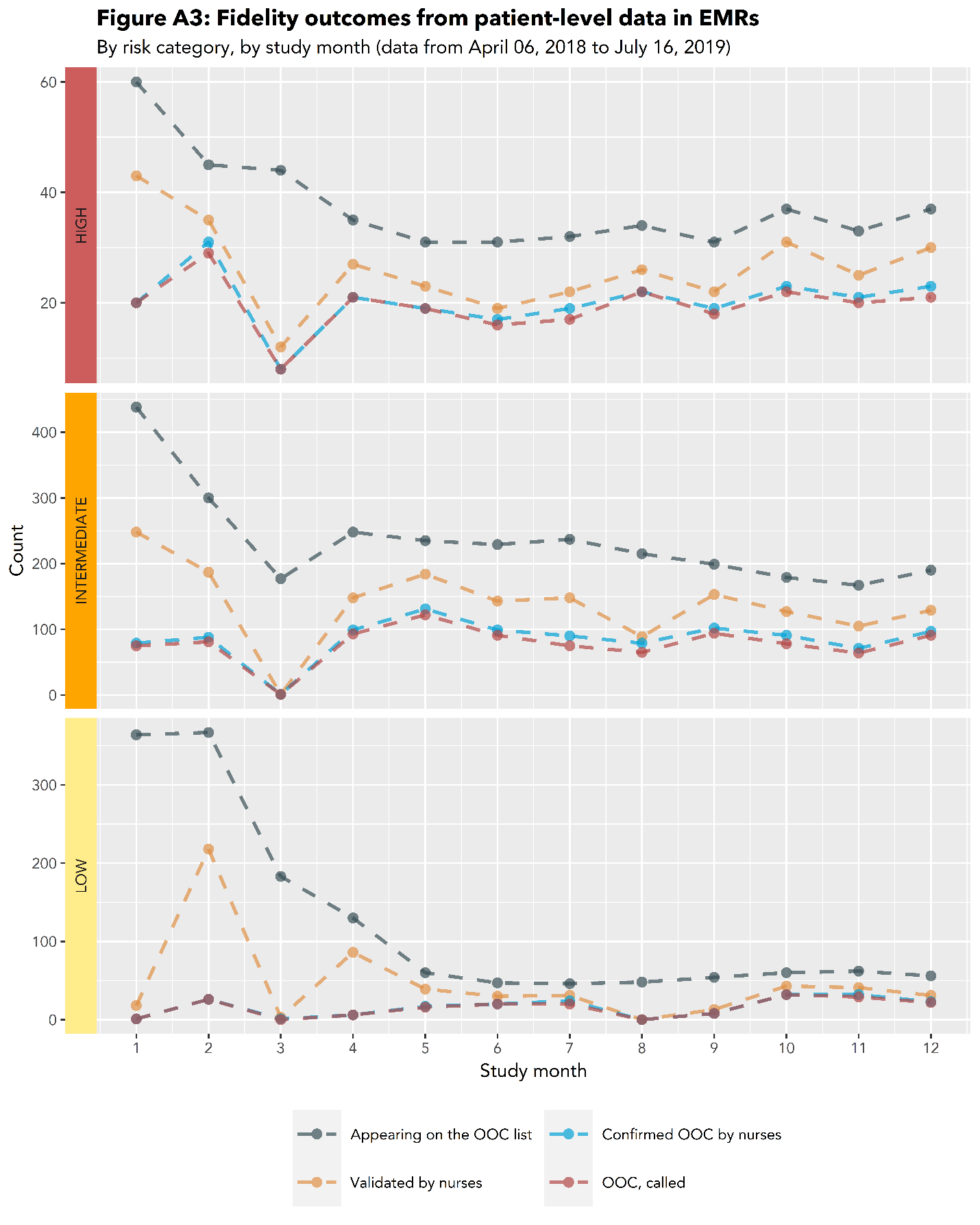

Supplement: Supplementary file 3 — Supplementary file3 (DOCX 2885 KB) [file 10461_2022_3888_MOESM3_ESM.docx]
