# Supplementary material for: Implementation of Lost & Found, An Intervention to Reengage Patients Out of HIV Care: A Convergent Explanatory Sequential Mixed-Methods Analysis
Source: AIDS Behav. 2022 Oct 22;27(5):1531–47. doi: 10.1007/s10461-022-03888-y (PMC10130100; doi:10.1007/s10461-022-03888-y)
Supplement: Supplementary file 5 — Supplementary file5 (DOCX 20 KB) [file 10461_2022_3888_MOESM5_ESM.docx]

**Supplementary material 5:** Identified determinants and their impacts on implementation outcomes.

| **Implementation outcome*** | **Determinant**ꭝ | **Valence** | **Identified influence on implementation outcome(s)** | **Explanation(s)** | **Illustrative quote(s) or events** |
| --- | --- | --- | --- | --- | --- |
| Feasibility | Nature of the behaviour | Barrier | Lower feasibility scores in pre-implementation and early implementation. | Time-consuming task of addressing the long OOC list in early implementation. | “Assuming in the beginning, there’ll be a long list of patients, we probably won’t be able to get through them all right away.” – Nurse 1, pre-implementation |
|  | Information systems | Facilitator | i) Increase in feasibility of the OOC list from pre-implementation to month 1. ii) General increasing trend in fidelity (use of the OOC list) | i) Modifications to RISQ helped make reengagement easier and more thorough. ii) Gradual improvements to RISQ made the OOC list easier to use. | i) “We’ll be able to [identify patients] more systematically. We’ll be able to catch more that otherwise might fall through the cracks.” – Nurse 1, pre-implementation  ii) The study coordinator worked with nurses and the database manager to improve RISQ. |
|  |  | Barrier | i) Generally lower feasibility scores for the OOC list in early implementation, including a decrease in month 2. ii) For fidelity, decrease in patients validated in month 8; and, use of HIV follow-up tab only "some of the time". | i) Unintended or sub-optimal functioning of information systems, which impacted how patients were identified as OOC. ii) Two nurses were unable to access RISQ in month 8; and inherent limitations of RISQ required nurses to search other databases for patient information. | i) Changes to how HIV care visits were defined just prior to the month 2 focus group, suddenly increasing the length of the OOC list and nurses' workload. ii) Hospital server upgrades prevented access to RISQ for two nurses on remote desktops (new computers were eventually installed). |
|  | Assistance for organisational changes | Facilitator | Gradual increase across implementation outcome scores with a gradual decrease in variability throughout implementation. | The study coordinator (internal facilitator) helped address issues identified by various implementation stakeholders, particularly in early implementation where more adaptation occurred. | “[The study coordinator] is responsive and answers quickly. If there’s a solution, there’s usually something that’s found pretty quickly.” – Nurse 1, Month 3 |
|  | Compatibility | Facilitator | High pre-implementation feasibility scores for phone calls. | Culture of the clinic; nurses' views that monitoring and reengaging OOC patients were central to their responsibilities; and, nurses' previous efforts to call patients perceived as potentially OOC, prior to the Lost & Found study. | “We were already doing phone calls before; it’s part of the job. It’ll just be easier to do than before.” – Nurse 2, Month 6 |
|  | Clarity | Barrier | Decreased in feasibility of phone calls from pre-implementation to month 1. | Uncertainty about how frequently patients should be called. | “I’m calling the same patient 3, 4 times, every week or every second week, when I decide to give them a break .” – Nurse 2, Month 1 |
|  | Availability of necessary resources | Barrier | i) Lower feasibility scores throughout implementation. ii) Lower self-efficacy (adoption subscale) in months 1 and 2 of implementation. iii) For fidelity, decreases in the number of patients validated, confirmed OOC, and called in months 1 and 3. | i) Limited or variable human (nursing) resources. ii) Nurses’ concerns about their ability to book patients in with doctors. iii) Occurred when any nurse, especially Nurse 2, was sick or went on an extended vacation. | i/iii) “If one of us is on vacation, even it’s not [Nurse 2], there is only three of us. Then we’re busy enough with doing just the clinic stuff that we won’t necessarily have time to do it.” – Nurse 3, Month 12 ii) “Summer is coming so it’s been harder now because, for most doctors, the next availability is in [two months]. The longer it takes for an appointment, the less chance we have for the patient to show.” – Nurse 2, Month 2 |
|  | Referral processes | Barrier | Lower feasibility scores throughout implementation. | Limited MD appointment availability made patient reengagement more challenging. | “I hear [Nurse 2] say it often like: ‘I don’t want to even phone this person because I don’t have any [MD] appointments to offer them.’” – Nurse 1, Month 12 |
|  | Patient behaviours, needs, and preferences | Barrier | Lower feasibility scores for the phone calls compared to managing the OOC list throughout implementation. | Challenges that patients face in their lives (i.e. competing priorities and essential needs) which complicate patient reengagement; language barriers; and nurses' concern about making patients feel confronted or guilty about their absences from care. | “If they live far, if they have to ask to have time off, [… if they have to] get home in time to pick up kids from school, it constrains people into a small window that they can come to the clinic.” – Nurse 1, Month 6\| “When I meet them, I’m trying not to confront them. I’m trying to be positive.” – Nurse 4, Month 2 |
| Acceptability | Observability | Facilitator | Increased acceptability for the OOC list throughout implementation. | Nurses appreciated being able to have a real-time OOC list that categorises patients into different risk categories. | “Little nicks in [the database] but generally it’s good. It’s kind of exciting to see when it works. When it pops up a name at the top and you say: ‘Oh, yes, that’s definitely an [out-of-care] patient’” – Nurse 1, Month 1 |
|  | Source of the recommendation | Facilitator | High acceptability scores throughout pre-implementation and implementation. | Nurses were central in developing the Lost & Found intervention, and drew upon their previous reengagement efforts to do so. | “I’m looking forward to it. We’ve been asking for something. We’re always doing it anyways but hopefully with the tool, it’ll be more systematic.” – Nurse 1, pre-implementation |
| Adoption | Domain knowledge | Facilitator | Increase adoption scores throughout pre-implementation and implementation. | Nurses understood many of the barriers that patients face in maintaining engagement in care, and could differentiate those truly OOC from those prematurely flagged by RISQ. | “There [are] so many patients that we could quickly remove just by looking at the list: ‘Oh, he’s at another clinic, he’s there, he’s there.’” – Nurse 2, pre-implementation |
|  | Skills needed to adhere | Facilitator | Increase adoption scores throughout implementation. | Nurses have the skills necessary for engaging with patients on the phone and upon reengagement. | “[Patients] don’t know [nurse 2], but when she calls, she’s open and people feel a little more inclined to want to come back.” – Nurse 1, Month 6 |
| Fidelity | Knowledge of own practice | Facilitator | Facilitated fidelity to Lost & Found throughout implementation. | Nurses could overcome inherent limitations related to the RISQ database and pull information about patients from other sources, such as health registries and other systems; they could apply their knowledge in phone calls with patients. | “[The phone calls] entail discussing medical issues that [others] won’t necessarily be aware of, they don’t understand.” – Nurse 2, Month 12 |
|  | Team processes | Facilitator | Facilitated fidelity to Lost & Found throughout implementation. | Nurses collectively managed their responsibilities, including Lost & Found; and nurses engaged other members of the clinical team who provided positive feedback and support. | “We have different responsibilities. I think we manage to work it out amongst each other. There’s no clear division, we try to help each other.” – Nurse 2, Month 12 |
| *Refers to the implementation that the given determinant will impact directly; it will also indirectly impact implementation outcomes below the given outcome.  ꭝDeterminants from the TICD framework, identified in nursing focus groups. | | | | | |
