# Supplementary material for: Implementation of Lost & Found, An Intervention to Reengage Patients Out of HIV Care: A Convergent Explanatory Sequential Mixed-Methods Analysis
Source: AIDS Behav. 2022 Oct 22;27(5):1531–47. doi: 10.1007/s10461-022-03888-y (PMC10130100; doi:10.1007/s10461-022-03888-y)
Supplement: Supplementary file 6 — Supplementary file6 (DOCX 1681 KB) [file 10461_2022_3888_MOESM6_ESM.docx]

Supplementary material 3: Clinical EMR Version history

The following document provides a detailed account of the Lost & Found related functionalities of the clinical electronic medical record (EMR) at the Chronic Viral Illness Service of the McGill University Health Centre (CVIS-MUHC), Montréal, Canada. Details include the original request, different versions, and changes at each iteration.

Notably, in this document, the acronym LTFU refers to “lost to follow-up”, which is equivalent to the term “out-of-care” (OOC) is used in the published study results. The name of the clinic’s EMR is “RISQ”, which is how it is referred to throughout this document.

**Table of Contents**

[**Version history**](#_Version_history) 1

[v1.11](#v1_11)2

[v1.10](#v1_10)2

[v1.9](#v1_9)3

[v1.8](#v1_8)4

[v1.7](#v1_7)5

[v1.6](#v1_6)5

[v1.5](#v1_5)6

[v1.4](#v1_4)6

[v1.3](#v1_3)6

[v1.2](#V1_2)7

[v1.1](#V1_1)7

[v1.0](#V1_0)8

[v0.2](#v0_2)8

[v0.1](#v0_1)9

[Initial request 1.1](#InitialRequest1_1)10

[Initial request 1.0](#InitialRequest1_0)11

[**Appendix 1: LTFU-RPT versions**](#_Appendix_1:_LTFU-RPT) 16

[**Appendix 2: Option trees for pop-up menus**](#_Appendix_2:_Option) 19

[**Appendix 3: LTFU status bar functionality**](#_Appendix_3:_LTFU) 28

[**Appendix 4: Risk category heirarchy in RISQ**](#_Appendix_4:_Risk) 29

[**Appendix 5: Photos for specific errors**](#_Appendix_5:_Photos) 31

[**Appendix 6: Order of the LTFU list**](#_Appendix_6:_Order) 32

# **Version history**

| **RISQ version** | **Tab**  **Version** | **Install Date** | **Changes/Features** |
| --- | --- | --- | --- |
| 2.58 | v1.11 | 2019-03-26 | - Addition of “Mother to child transmission” to the INTERMEDIATE risk category (*email 2019-02-20)*   - Updated RPT in [Appendix 1](#_v1.10) - Added option "Unspecified date for walk-in" for “Arranged appointment/walk-in" and related functionality in the contact/validation attempts pop-up *(email 2018-12-19)*   - Users can now click a check box for “Unspecified date for walk-in” and enter a “Next attempt date” when they arrange a potential walk-in with a patient.     - Gives nurses and patients more flexibility for situations where nurses provide a range of possible walk-in dates     - Before, they could only add in a visit or select an existing visit.   - Patients will be LTFU (pale) or not (white) depending on their answer to the “Is this patient LTFU” question. - Wording changes *(email 2018-12-19)*   - In the “Result” column of “Contact and validation attempts”, it will now read "Contacted, upcoming HIV care visit" instead of "Contacted, upcoming appointment"   - "Were you able to contact the patient?" changed to "Were you able to contact and speak directly with the patient?" in the contact/validation pop-up box     - For the response options, "Contacted the patient" changed to "Spoke directly with the patient" and "Could not contact the patient" changed to "Did not contact or speak directly with the patient" |
| 2.53-2.57 | Not installed or not related to L & F | | |
| 2.52 | v1.10 | 2018-11-08 | - Risk category calculation is now run automatically every evening *(email 2018-09-06)*   - Previously done ad hoc by research coordinator. - Correction of the “two-month rule”   - LTFU status put to “no” rather than to “yes” in the automatically entered contact/validation attempt     - Developer was mistakenly told to set to “yes” on 2018-10-11 by research coordinator. - Clarification for re-calculation of risk categories *(email 2018-11-2018)*   - Risk category recalculated after *either* a new VL or CD4 that results in a higher risk category     - vs. both in v1.9   - Correction of HCV history risk category calculation (from v1.9) - Wording changes (after launch of v1.10)   - "On vacation" option in "Planned or unavoidable CVIS care interruptions" to "Out of country/on vacation" (changed 2019-03-15)   - "Arranged appointment/walk-in" from "Booked for appointment/walk-in" in contact outcomes (Changed 2018-12-17)   - "Arranged for outreach nurse to contact patient" added to non-contact outcomes (changed 2018-12-14)   - "Unable to re-engage OR Temporary transfer of care" from "Planned or unavoidable CVIS care interruption" (changed 2018-12-13)   - "Other priorities than HIV care OR Not ready to re-engage" from "Unwilling to engage in care" (changed 2018-12-13) |
| 2.51 | Test version only | | |
| 2.50 | v1.9 | 2018-10-31 | - New patients without HIV are automatically classified as “Not followed: No HIV” *(email 2018-09-06)*   - When the patient is created in RISQ - Changes in HIV status (due to data entry errors) result in automatic changes to follow-up status *(email 2018-09-06)*   - For changes from “No HIV’ to “HIV”, the patient will remain “Not followed” (if already classified as such).     - A new visit will result in the patient being switched from “Not followed” to “Followed”, if necessary.   - For changes from “HIV” to “No HIV”, the patient’s HIV follow-up status will change form “Followed” to “Not followed”, if not already the case.     - A note “No HIV" will be added in the notes. - Corrections to the risk category calculation, considering manual entries and old information *(email 2018-09-28)*   - Note: This is a correction to meet was is described in v0.1.   - For manual entries only, recalculation of risk category is only done if events (labs, new medications, etc.) occur *after* the start date of the manually entered risk category.     - - For example, people will only be reclassified if there is a new VL and CD4 after the start date of the current risk category, that would bring the patient into a higher risk category.     - The “CD4<200” and “Youth” arguments in the INTERMEDIATE category are ignored after a manual entry.     - HCV tests will only be ignored if they had a positive HCV test before the start date of the risk category. Otherwise, it can be assumed they caught HCV, and the risk category will be recalculated. *(email 2018-10-19)*       - Note: not functional in this version. Correction in v1.10.     - New non-ARV medications will only be considered if they are entered after the start date of the risk category, and have an end date that is missing or after today’s date (i.e. active medications only). (*email 2018-10-22)*   - Thus, manually decreased risk categories won’t be automatically overwritten by a higher risk category every time the risk category calculation is ran     - - Allows nurses to input lower risk categories, which won’t be changed until the patient comes back and has a new, higher risk event. - Addition of a “two-month rule” for the INTERMEDIATE and LOW risk categories. *(email 2018-09-28)*   - All INTERMEDIATE and LOW risk patients with visits booked on their own within two months of being marked LTFU by the system are automatically classified as “Not LTFU” until the date of their latest CVIS visit within the two months following being marked as LTFU.     - Note: Research coordinator mistakenly told developer to classify patients as “LTFU” if they have visits within two months, on 2018-10-11. Corrected in v1.10.   - Four scenarios when an INTERMEDIATE or LOW risk patient is marked as LTFU or not:     1. Patient has no visits within the two-month window and no active contact/validation attempts 🡪 no new automatic contact/validation entry (i.e. still LTFU)     2. Patient has one or more active contact/validation attempts, regardless of visits within the two-month window 🡪 no new entry (i.e. LTFU or not depending on what was in the contact/validation attempt)     3. Patient has a contact/validation attempt created less than 61 days ago and no active contact/validation attempts, regardless of whether or not they have a visits within two months of being marked LTFU 🡪 no new entry (i.e. still LTFU)     4. Patients has no active validation/contact attempts, their most recent contact/validation attempt was over 61 days ago, and they have a visit within two months of being marked as LTFU 🡪 System creates a new contact/validation attempt with a next attempt date that corresponds to the last visit within the two month window (i.e. not LTFU)        - See note above for mistake corrected in v1.10.   - Helps remove patients who are managing their own follow-up (but a bit behind schedule) from the LTFU list.   - HIGH risk patients are not included in this rule. - Reason “No HIV” added to list of “Not followed” reasons in “Follow-up status” pop-up *(email 2018-10-15)* - Wording changes:   - In the “Follow-up status” pop-up box, "Is this patient being followed at the CVIS?" changed to "Is this patient being followed for HIV care at the CVIS?" |
| 2.49 | v1.8 | 2018-08-14 | - Clicking “calculate risk category” now hands off the risk category calculation to the system. *(email 2018-08-03, follow-up 2018-08-08)*   - The risk category assigned by the button is considered an automated change     - “Changed by” is written as “System ([USER])”     - This means that patient’s risk category could be moved in either direction (either higher or lower), vs. the risk category calculation done for the overall patient sample, which can only increase the risk category after a manual change. (per [diagram in Appendix 4](#_V1.2_and_later:))   - However, if the person clicks the button THEN changes the category to something else within the pop-up (or changes the reason), the change will be considered a manual change.     - “Changed by: [USER]” - Resolution of an unfixed issue with risk categories being changed to “UNKNOWN” from v1.7 and patients being recategorized from higher risk categories to lower risk categories in the system.   - For the UNKNOWN issue, works as described in v1.7 & v1.2   - For he recategorization issue, works as described in v0.1 |
| 2.48 | v1.7 | 2018-08-07 | - New criteria for the HIGH risk categories *(email 2018-07-16)*   - All new patients are now classified as HIGH when created in RISQ for the first time   - Will be considered a manual change     - “Changed by” will be listed as “First entry” - Improvements to risk category calculations *(email 2018-07-10)*   - All patients at least a CD4 and VL will be given a risk category other than UNKNOWN     - Previously only CD4s and VLs that occurred after the start date of the UNKNOWN risk category were considered.       - Thus, patients with new measures     - Now this logic only applies non-UNKNOWN categories.   - NOTE: This did not work. Fixed in version v1.8 - Calculate risk category button *(email 2018-08-03)*   - When button is clicked, the patient’s risk category will be calculated per the LTFU-RPT.   - If VL and/or CD4 are missing, no risk category will be assigned and an error message will be displayed.   - This calculation will not take into consideration the previously manually entered risk category. - Added “How the risk category is calculated” button *(email 2018-08-03)*   - Sends you to a folder in the share drive, where a document explaining the risk category will be present.     - Vs. a direct link to a document, which would have to be named and sent to Alex. |
| 2.47 | v1.6 | 2018-07-18 | - Automatic updates to “Follow-up status” for new appointments   - When patients have a new visits entered, or previously entered upcoming visits changed to “attended”, their follow-up status is automatically changed to “Followed: New appointment” *(email 2018-Jun-22)*     - People without HIV are ignored.     - New labs have no effect. - Issues with “Langues préférées” box solved on 2018-07-17   - Was producing an error message when information was entered. *(email 2018-07-17)* |
| 2.46 | v1.5 | 2018-07-12 | - Addition of the question “In your opinion, is this patient lost to follow up?” in the “Contact attempts and validation” pop-up box *(email 2018-05-28)*   - Yes/No check boxes   - If the user selects “Yes” everything works as normal (pale colours, etc.)   - If the user selects “No”, user will see a message stating “Please state your reasoning in the notes section” and will be unable to save the message unless they do so.     - The patient will be labelled “Engaged in care” and removed from the LTFU list.   - A new column “LTFU?” was added to the “Contact attempts and validation” table, which documents the users’ response.   - Pop-up box flow in [Appendix 2](#_v1.5). |
| 2.45 | v1.4 | 2018-07-06 | - Order of the LTFU list reorganized per OrderOfLTFUList_2018May30.xlsx *(email starting 2018-05-29)*   - Grey section organized as 1. HCV 2. None 3. HIV 4. Co-Infected 5. Mort (Fleur)   - Rather than being classified as grey in the LTFU list, “HCV” and “None” infection types are coloured in white (indicating that they could be followed up for non-HIV care). - New column “Care type” on the LTFU list.   - Each patient labeled with a numeric code corresponding to their infection type - 0 = None; 1 = HIV; 2 = Co-infected; 3 = HCV *(email 2018-05-31)* - Column with LTFU status colours renamed from “LTFU” to “HIV-care LTFU” *(2018-05-31)* |
| 2.44 | v1.3 | 2018-06-12 | - Box “Langues préférées" added in the top left quadrant *(original email 2018-03-19, resent 2018-05-25)*   - Note: non-functional. Fixed in v1.6. - Colours of the LTFU changed *(email 2018-05-18)* OrderOfLTFUList_2018May18.xlsx)   - Risk category colours changed to HIGH, INTERMEDIATE, and LOW   - Colour of the UNKNOWN category changed as well.   - Updated RPT in [Appendix 1](#_v1.10) - Automatic classification of deceased patients as “Not followed” *(Email 2018-05-31)*   - When death entered in the history tab, patient’s follow-up status automatically changed to “Not followed”   - When follow status is changed to “Not followed: deceased” the information about the death is added to the history tab. - VLs and CD4s are no longer considered as attended visits *(email 2018-05-23)*   - Only visits with the check box ticked for “Attended at the CVIS” are considered attended visits.   - I.e. removing change made in v0.2   - Updated RPT in [Appendix 1](#_v1.10) - Improved handling of LTFU status based on manual risk category changes and missing values *(email 2018-05-18)*   - If patient has any attended visits and a risk category that isn’t UNKNOWN, the LTFU status (“LTFU” or “Engaged in care”) will be determined based on the risk category and the number of days since the most recent visit.     - Before, the LTFU status would be “unknown” unless all of VL, CD4, and Visits were present, even with risk category manually entered.       - I.e. for patients without the lab information, their LTFU status would remain “unknown”, even though the necessary information for their LTFU status was available. - Bug fixes for an issue concerning displaying non-contact outcomes in the “Contact attempts and validation” table. *(email 2018-05-23)* |
| 2.43 | v1.2 | 2018-05-17 | - Order of the LTFU changed from longest to shortest absence, by category, to reflect original proposal *(email 2018-05-09)*   - Vs. alphabetic order of doctor’s name - Name of “Attended?” check box changed to “Attended at the CVIS?” for better specificity *(email 2018-04-20, response 2018-05-11)* - Printing functionality for LTFU list added - Improved handling of manual risk category entries   - *NOTE:* Not functional. Not addressed until v1.8 (2018-08-14)   - Addition of UNKNOWN in the hierarchy *(email 2018-05-11)*     - Manually entered risk categories take precedence over lower categories *and* UNKNOWN       - E.g. manually entered HIGH won’t be changed to INTERMEDIATE or LOW or UNKNOWN, unless done manually.       - [Diagram in Appendix 4](#_V1.2_and_later:) explains the hierarchy visually. - Added functionality to the “Contact and validation attempts” pop-up box   - “Care interruptions” functionality added *(email 2018-04-06)*   - Pop-up box flow in [Appendix 2](#_v0.3). |
| 2.42 | v1.1 | 2018-05-03 | - Solved issues with viewing entries   - Can double click entry or click a button to view “Risk-category history” or “Follow-up status” *(email 2018-04-20)* - Text wrapping added to the notes in the pop-ups *(email 2018-04-20)* - Improved functionality of the “Missed visits” table. Only CVIS services considered. *(email 2018-04-20)* Specifically:   - Outpatient   - Medical clinic without appointment   - Nurse   - Psychologist   - Social worker   - Pharmacist   - Psychiatrist   - Participating in Study   - Annual Nursing Evaluation   - Day Hospital   - Other - Documentation of “Reasons” column of the “Risk category history” table improved. *(email 2018-04-20)*   - For a given patient, only criteria met for the assigned category are included.     - E.g., for a patient in the INTERMEDIATE risk category due to their age and HCV history, only “Youth” and “History of HCV” would be displayed, not their CD4 or VL (since those would be for the green risk category).   - Ran one-time script to fix reasons that were not manually entered. - Bug fixes   - RISQ was crashing when specific options were in “Contact and Validation attempts”. *(email 2018-05-01, photo in* [*Appendix 5*](#_V1.1)*)*.   - Position of the “Risk category history” title corrected     - It was right aligned before *(email 2018-04-20)* |
| 2.41 | v1.0 | 2018-04-19 | - LTFU status in the LTFU list for a given patient now representative of their LTFU status bar   - Ordering of LTFU list according to the table in [Appendix 6](#_v1.0) (*email 2018-04-16)*     - Dark colours, for patients LTFU with no active contact/validation attempts     - Pale colours, for patients LTFU with an active contact/validation attempts     - Beige, for engaged patients (or patients without HIV)     - People with missing VLs or CD4s given the risk category “Unknown” and flagged in orange at the bottom of the LTFU list (below the engaged) - Some optimisations of the risk category calculation. |
| **BETA VERSIONS (Limited functionality)** | | | |
| 2.40 | v0.2 (Beta) | 2018-04-12 | **Changes:**   - “Recent CD4” and “Recent VL” boxes added to Top right quadrant of HIV follow-up tab *(meeting with developer, 2018-03-23)* - “Last appointment” box renamed to “'Last visit, CD4, or VL” *(email 2018-04-06)*   - Last VL and/or CD4 included in the box - CD4 and VL dates considered as attended visit dates in the calculation of LTFU status *(email request 2018-04-06)*   - In other words, if someone has a VL listed 3 months ago, but a last visit 9 months ago, consider the VL date as the most recent visit.   - Updated RPT in [Appendix 1](#_v1.10)   - Note: removed in v1.3 (2018-06-12) - Risk category calculation fixes   - History of HCV calculation included (*Email 2018-04-06*)     - 'Hepatitis C RNA' (code 31) used for confirming “History of HCV”   - Polypharmacy calculation included (>=5 non HIV meds) - Improved handling VL values, codes, considering missingness, odd formatting:   - Missing tests not displayed in the 'Last visit, CD4 or VL' box *(email sent 2018-04-09, response 2018-04-12)*   - Missing tests not used in the calculation of LTFU status or risk category. *(email 2018-04-12)*   - Most recent *valid* VL is taken, the invalid entries are ignored. *(email 2018-04-12)*   - Missing VL still displayed in the 'Recent VL' box *(email 2018-04-12)*   - If the *code* for the VL is equal to 50, the VL is ignored (data entry error). *(email 2018-04-12)* - Added options to the “Contact and validation attempts” pop-up box   - “Left message” now available *(email 2018-04-06)*   - Pop-up box flow in [Appendix 2](#_v0.2). - “Add shared care arrangement” text removed, since no functionality was assigned. *(email 2018-04-06)* - Formatting issues corrected in LTFU status bar   - Space added after “Care interruption:” *(email 2018-04-06)*   **Missing functionalities:**   - Still no calculation of pale colours in the LTFU list, due to a slow calculation. The colours have been applied for the current set of patients. |
| 2.39 | v0.1 (Beta) | 2018-04-06 | **Missing functionalities from request:**   - No statement “Please provide a reason for their unwillingness, if possible, in the notes section.” For unwilling to engage in care.   - Not implemented going forward. - No automatic import of visits from OACIS - Limited functionalities in the “Contact attempts and validations” pop-up box   - No 'Care interruptions' functionalities in the "Contact attempts" pop up box   - No options displayed when “Could not contact patient” was selected (i.e. Left message, etc.)   - Pop-up box flow in [Appendix 2](#_v0.1). - No capabilities for printing the LTFU list - No automatic calculation of risk category. - No “history of HCV” calculation for risk category - No “polypharmacy” calculation for risk category - Pale colours of RISQ categories not present, still being worked on by the developer.   - Basic risk category only (i.e. red, yellow, green, with beige for all other scenarios.)   **Changes**   - “Medical examinations” changed to “Visits” - Change to titles for different non-LTFU scenarios. Now “Care interruption: [Reason]”, or “Not followed: [Reason]” - Tab renamed “HIV follow-up” - Improved logic for calculating risk categories, given lab dates (*meeting with developer, 2018-03-23)*   - Manually entered risk categories will only be overwritten by higher risk categories (i.e. if risk category set at LOW, risk category will be changed to INTERMEDIATE or HIGH based on new information.     - See diagram in [Appendix 5](#_V0.2_and_later:), created in email sent 2018-Jul-17     - *NOTE:* This was not properly implemented until v1.8 (2018-08-14),       - i.e. patients were reclassified from HIGH (set manually) to INTERMEDIATE or LOW (by the system, whenever a calculation was run).   - Only information after the start date of the risk category will be considered.     - I.e. labs with dates after the start date     - Note: Not properly implemented until v1.9 (2018-10-31) |
| **PRE-DEVELOPMENT** | | | |
| NA | Initial request 1.1 | 2018-01-17 | **Changes:**   - “Follow-up” tab moved to the fifth position, rather than second, in the list of tabs. - More clarity added surrounding the functionality of LTFU status bar in the Follow-up tab [(Appendix 3, Initial request v1.1)](#_Initial_request_V2.0), for the developer.   - Slight change in colours 🡪 *All* patients who are LTFU and have validation/contact attempts will be in the pale colours until the next attempt date or re-engagement.     - Whereas before, people who had upcoming appointments would be labelled in grey.     - People who are engaged will be labelled in beige (the colour of the other engaged patients) rather than grey. - More clarity surrounding the functionality of the risk categories within the risk category table [(Appendix 4, Initial request v1.1),](#_Initial_request_V2.0_1)  for the developer.   **Additional features:**   - **Changes to the “Visits” tab**   - Automatic import of Visits into RISQ   - Additional checkbox “Attended?” to document whether or not scheduled appointments have been attended.     - Will allow for the “Missed visits” functionality.   - The most recent upcoming visit will be displayed in the “Next scheduled appointment” box in the “Follow-up tab”   - Past visits where the “Attended” box has been checked will be displayed in the “Previous visits” box of the “Follow-up” tab   - Past visits where the “Attended” box has ***not*** been checked will be displayed in the “Missed visits” box of the “Follow-up” tab |
| NA | Initial request  1.0 | 2017-12-21 | PowerPoint document sent to developer, outlining the requested functionality of the RISQ tab.  **Included features:**  **LTFU List**   - LTFU list on opening page of risk, with LTFU patients categorized into High, Intermediate, and Low risk categories based on the triage step of the [LTFU-RPT](#_Initial_request_(2017-Dec-21)).   - Option to print the list (in case of manual monitoring). - Patients who are engaged in care are in beige coloured rows. - Pale colours correspond to patients who are LTFU but have been validated by nurses. This is explained further in the description of the [LTFU status bar.](#LTFUStatusBarInitialRequest) - Changes in the patient’s RISQ file, such as entries in the Follow-up tab or new visits, will be reflected in the LTFU list.   - If a LTFU patient comes in for a visit, they will be removed from the top of the list (i.e. coloured beige, since they are now engaged in care).   - If a nurse changes the patient risk category, the patient could be moved to the section on the LTFU corresponding to their risk category, or removed from the LTFU list if they no longer qualify as LTFU for their risk category.     - E.g. if a LTFU patient in the High risk category has been out of care for 4 months, a change to the Intermediate or Low categories would put them under the 6 and 12 month limits for LTFU, respectively, and they would no longer be considered LTFU.   **“Follow-up” RISQ Tab**   - New tab “Follow-up” (second on the list of RISQ tabs) where all information related to patient HIV follow-up can be documented. By section, this includes:   *Upper left quadrant*   - - The current follow-up status written within a large rectangle bar, coloured according to their current risk category or the pale version of the given risk category (explained later).   - Small box displaying the date of the last appointment, with text to the left “Last appointment:”   - Small box displaying the date of the next scheduled appointment, with text to the left “Next scheduled appointment:”   - Small box displaying the number of days since the last appointment, with text to the left “Next scheduled appointment:”   - Small box displaying the patient’s risk category, which is automatically calculated based on other information available in tables in RISQ, with text to the left “Need for follow-up:”.   - Text “Change risk category:” with a button to do so.   - Text “Validate and contact:” with a button to do so.   - Text “Add shared care arrangement:” with a button to do so.   - Text “Add planned or unavoidable care interruption:” with a button to do so.   - Text “This patient no longer requires follow-up” with a button to do so.   *Bottom left quadrant –* “*Validation and contact attempts”*   - “Validation and contact attempts” table with columns for columns for:   - Date – The date of the validation or contact attempt   - Contact info – Where the patient’s contact information was search and/or found.   - Result – The outcome of the validation and/or contact attempt (i.e contacted, not contacted, etc.)   - Next attempt date - The date of the next contact attempt.   - Changed by - The user who entered or changed the contact/validation attempt.   - Notes – Any notes pertinent to the contact/validation attempt. - Buttons next to the table allow for new entries or editing entries, respectively.   *Top right of the tab – “Visit history”*   - Two tables, which draw information from the “Visits” tab.   - *Previous visits* which displays recently attended HIV care visits.   - *Missed appointments* which displays missed HIV care visits.   *Middle right of the tab – “Follow-up”*   - Three long tables stacked one on top of the next:   - *Risk category history:* displays a list of changes made to the patient’s risk category, manual and automatic. Includes columns for:     - Risk category – the risk category assigned     - Start date – Start date of the risk category     - End date – End date of the risk category     - Reason – the reason(s) that the patient was classified into the given risk category.     - Changed by – The user (or system) who changed/entered the risk category   - *Planned or unavoidable CVIS care interruptions:* Displays manually entered care interruptions, such as hospitalizations or incarcerations. Includes columns for:     - Start date – Start date of the care interruption     - End date – End date of the care interruption     - Reason – the reason for the care interruption     - Changed by – The user who changed/entered the risk category     - Notes – Any notes pertinent to the care interruption   - *Status -*The patients’ current HIV follow-up status. Includes columns for:     - Status – the current HIV follow-up status, either “Followed” or “Not followed”     - Reason for change – the reason for the change in follow-up status     - Date – the date of the change in follow-up status     - Notes – any notes pertinent to the change in follow-up status     - Changed by – the user (or system) who changed the follow-up status.   - A button next to each of the *Risk Category History* and *Planned or Unavoidable Care Interruptions* tables allow users to add entries to the table.   - A button next to each of *Planned or Unavoidable Care Interruptions* and *Status* allows for existing entries in the table to be modified.   - Entries can e double clicked to see more information.   *Bottom right of the tab – “Contact information”*   - Two tables:   - *Phone numbers:* The same table found in the sociodemographic tab. Changes in the follow-up tab are reflected in the sociodemographic tab as well.   - *Emergency/alternate contacts:* A table for nurses to input emergency or alternate contacts for patients. - **LTFU status bar:**   - The bar in the Follow-up tab should present the current follow-up status of the patient. It should be:     - Red if the patient is LTFU and in the high risk category. Text will read “LTFU: arrange visit within 48hours”       - Light red if the patient has a contact or validation attempt marked as “unwilling to engage in care” or “Unreachable” with a “Next attempt date” after the current date.     - Yellow if the patient is LTFU and in the intermediate risk category. Text will read “LTFU: arrange visit within 2-4 weeks”       - Light yellow if the patient has a contact or validation attempt marked as “unwilling to engage in care” or “Unreachable” with a “Next attempt date” after the current date.     - Green if the patient is LTFU and in the low risk category. Text will read “LTFU: arrange visit within 4-6 weeks.”       - Light green if the patient has a contact or validation attempt marked as “unwilling to engage in care” or “Unreachable” with a “Next attempt date” after the current date.     - Grey if the patient is not LTFU, was contacted and booked for an appointment, was not contacted but has an appointment within a reasonable time frame, has a care interruption, or is not followed. Text will read “Engaged in care”, the text within the “reason” column of the planned/unavoidable care interruptions table, or “Not followed by the CVIS”. - **Risk category assignment:** Patients are assigned into one of three risk groups based on the LTFU-RPT ([Appendix 1, Initial request](#_Initial_request_(2017-Dec-21))).   - Patients are assigned automatically by the system after each visit, based on their most recent measures.   - Risk categories can be changed manually to decrease or increase contact frequency. - **Risk category pop-up box**   - A simple pop up box with three options (one for each risk category) with a notes box title “Reason for change” that the user has to fill out in order to make the change. The three options read:     - High (Patient followed Q3 months, seen within 48hrs when LTFU)     - Intermediate (Patient followed Q6 months, seen within 2-4 weeks when LTFU)     - Low (Patient followed Q12 months, seen within 4-6 weeks when LTFU) - **Planned or unavoidable CVIS care interruptions pop-up box**   - A simple pop-up box with the questions:     - “Please select a reason for the care interruption. This patient will not be marked as LTFU until the specified end date.” (Drop down)     - “Start date (If unknown, enter today’s date and leave note below)” (Date entry)     - “End date OR number of days” (Date entry/integer)     - “Notes (optional)” (Free text) - **Emergency/alternate contacts pop-up box**   - A simple pop-up box with the questions:     - “Name:” (Short free text)     - “Relationship to the patient:” (Short free text)     - “Phone number:” (phone number box)     - “Extension (optional)”     - “Is this person aware of the patient’s serostatus?” (Y/N) - **Contact and validation attempts pop up box**   - Different answer options will display depending on answers to previous questions. This flow with the questions and answers is displayed in [Appendix 2 (Initial request, Contact and validation attempts).](#_Initial_request_(2017-Dec-21)_1)   - Information related to other tables (i.e. visits, phone numbers, care interruptions) will be filled in automatically. (i.e. When booked for appointment/walk in is selected, a visit will be added to the visits tab). - **Follow-up status pop up box**   - The question and answer flow, including wording, is displayed in [Appendix 2 (Initial request, Follow-up status).](#_Initial_request_(2017-Dec-21)_2) - **Automatic changes to statuses based on updated information (visits and/or tests)**   - If a patient who is experiencing a care interruption (that has been documented in the Follow-up tab) comes for a visit before the manually entered end of the care interruption, the end of the care interruption will be changed to the date of the visit and the patient will be marked as “Engaged in care”.   - If a patient who has been marked as “not followed” comes in for a visit, their status will be changed to “followed” and they will be marked as “Engaged in care”.     - This doesn’t apply when the reasons for being “not followed” is death.   - If there is any death information in the “History” tab, the follow-up status will be changed to “Not followed” and the edit function for that table will be disabled. |

# **Appendix 1: LTFU-RPT versions**

***Note:*** The criteria that only patients who have had visits within the past five years has been removed here, since it is not applied automatically by the system. It was applied manually by the nurses and the research coordinator.

### [v1.11](#v1_11)


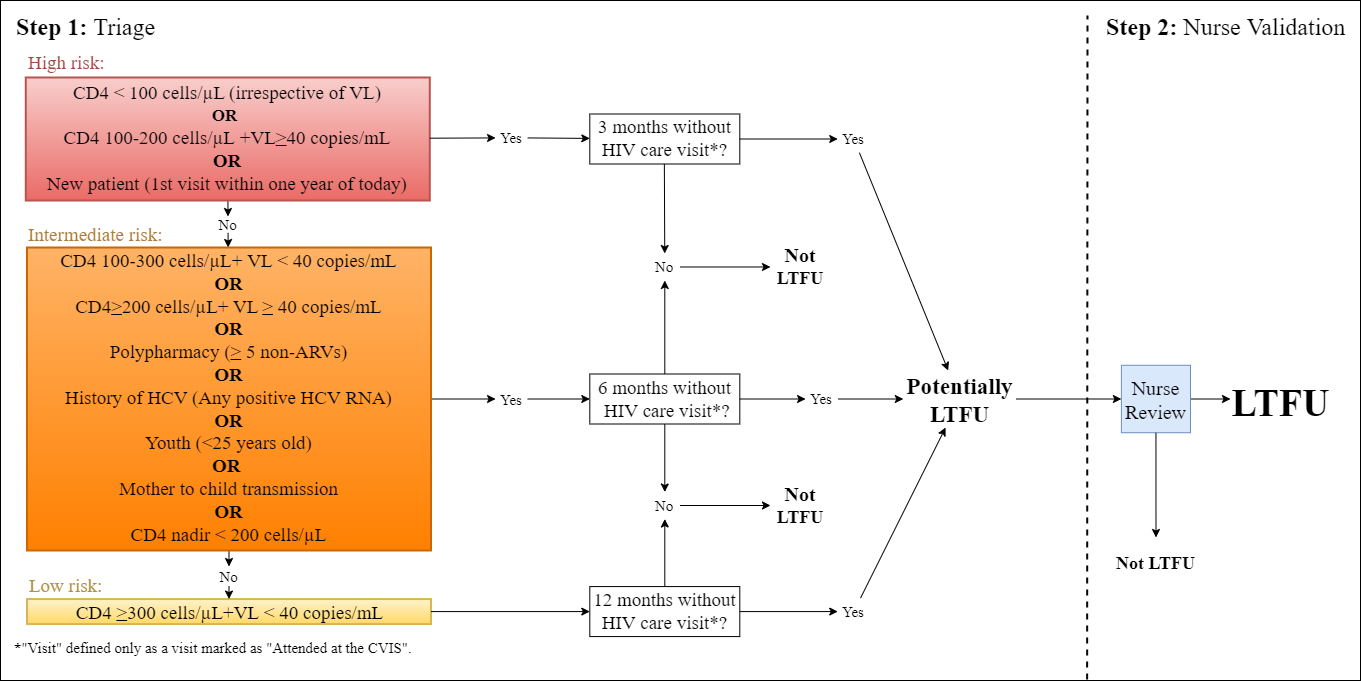


### [v1.7](#v1_7)


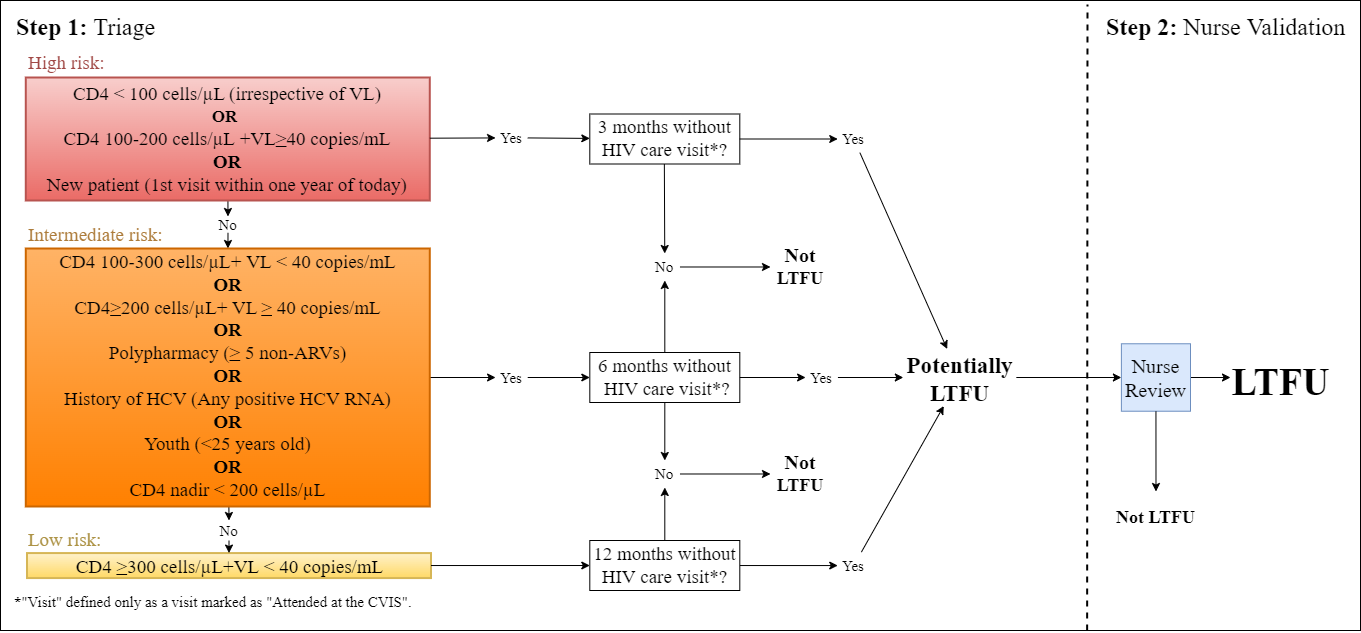


### [v1.3](#v1_3)


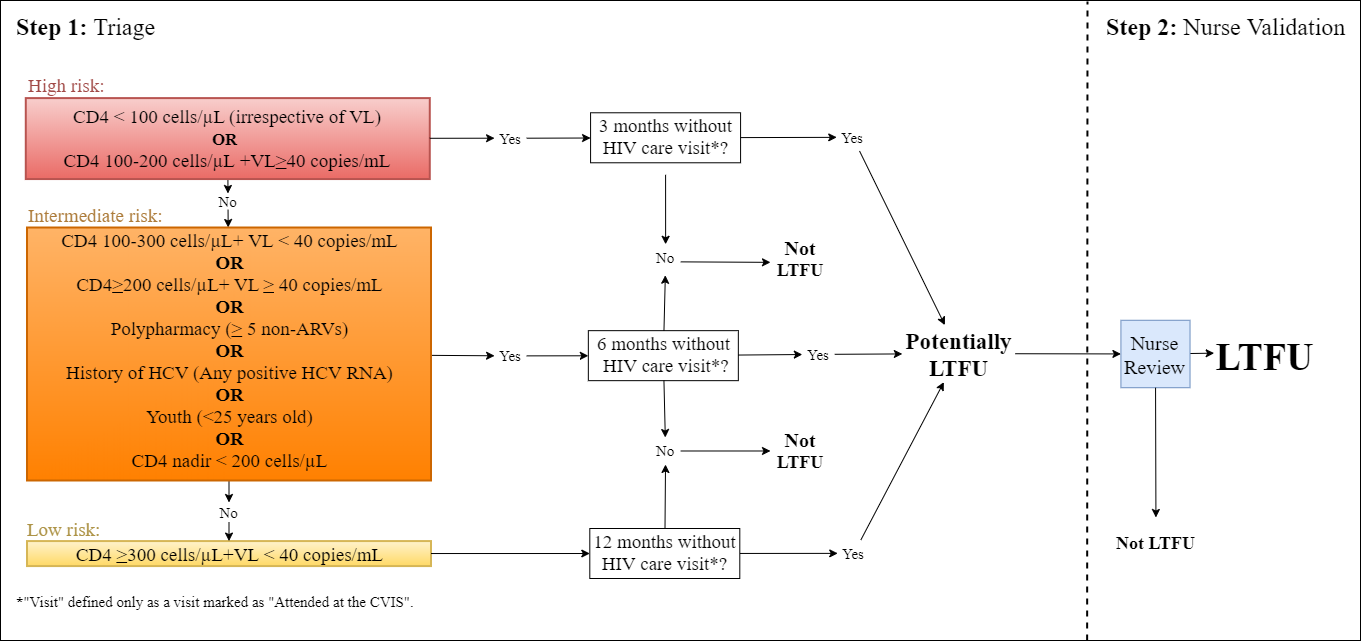


### [v0.2](#v0_2)


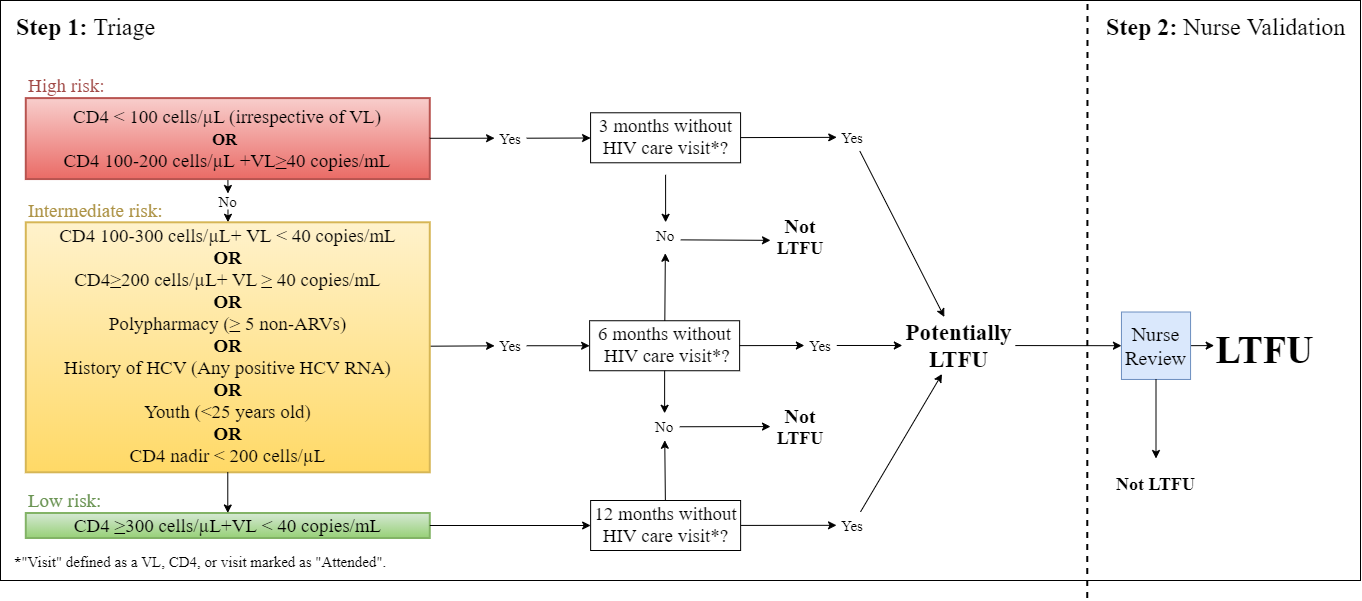


### **[Initial request](#InitialRequest1_0) 1.0 & 1.1**


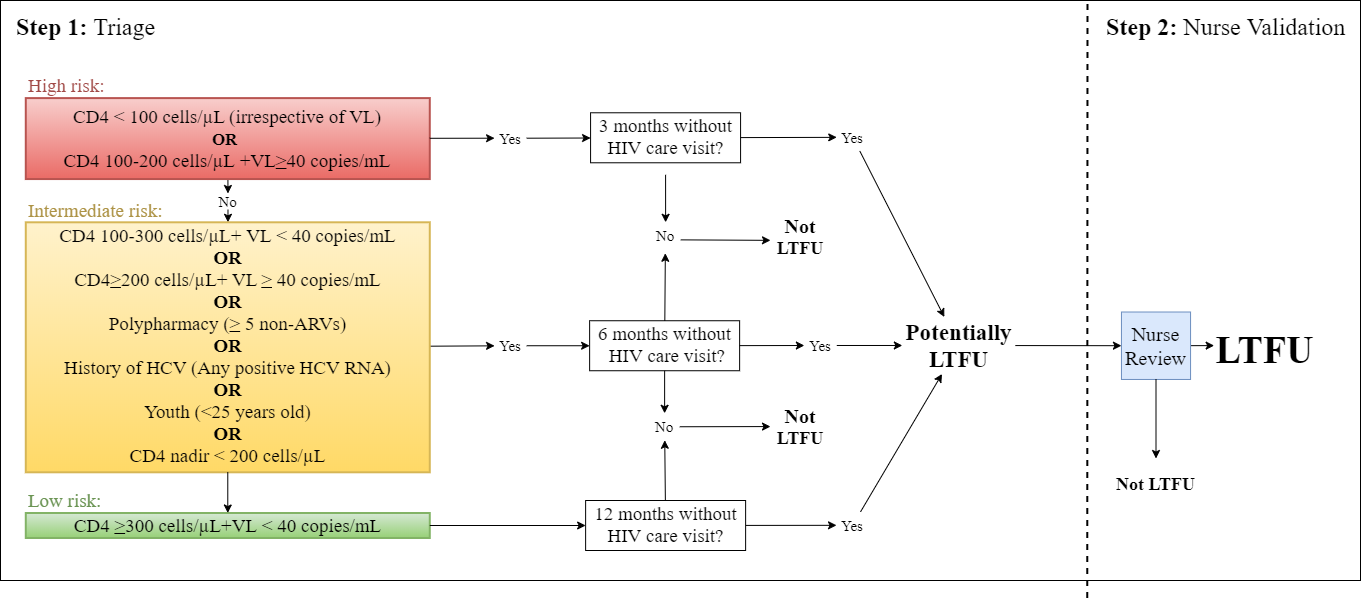


# **Appendix 2: Option trees for pop-up menus**

## **Contact and validation attempts**

###
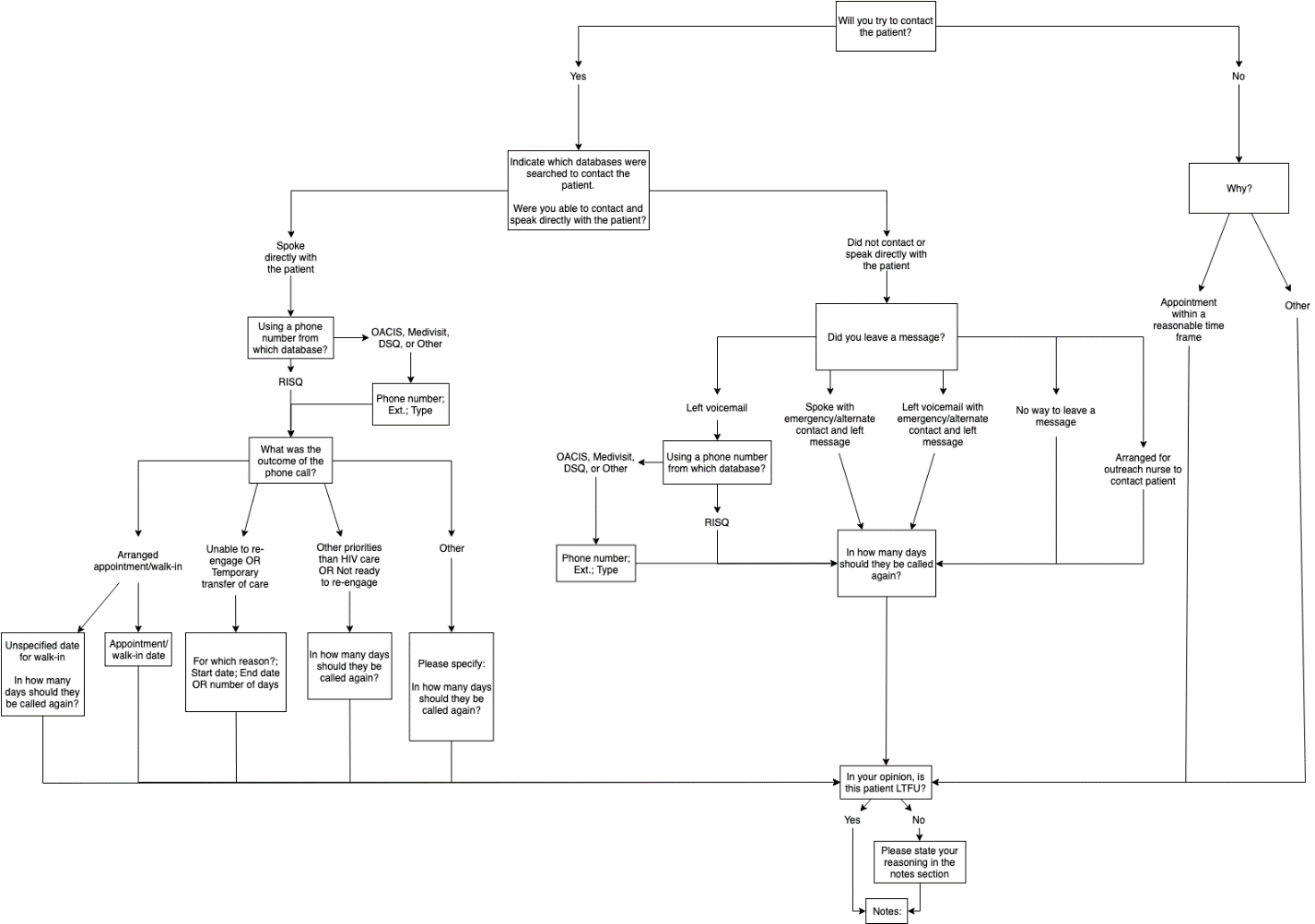
[v1.11](#v1_11)

###
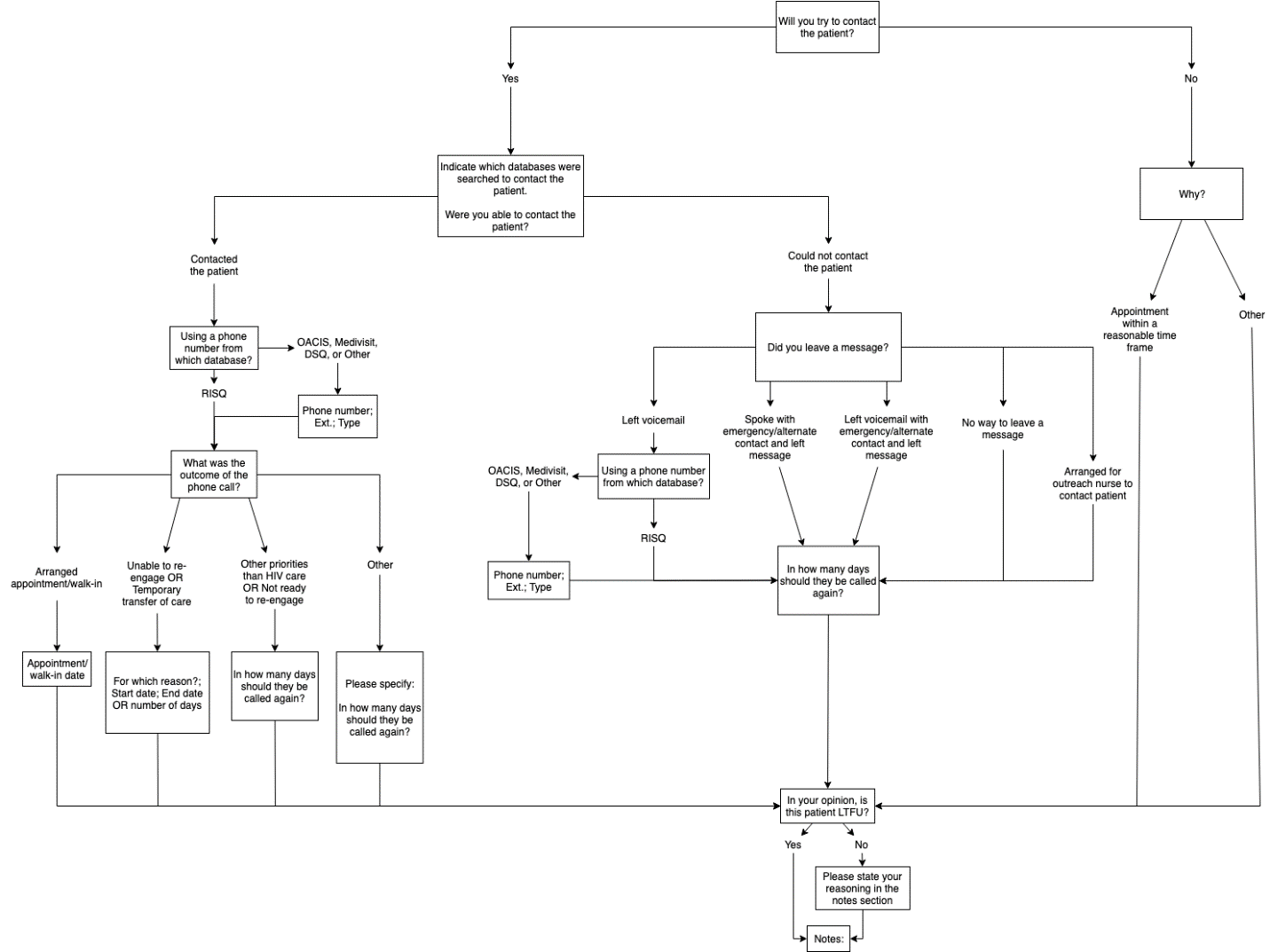
[v1.10](#v1_10)

###
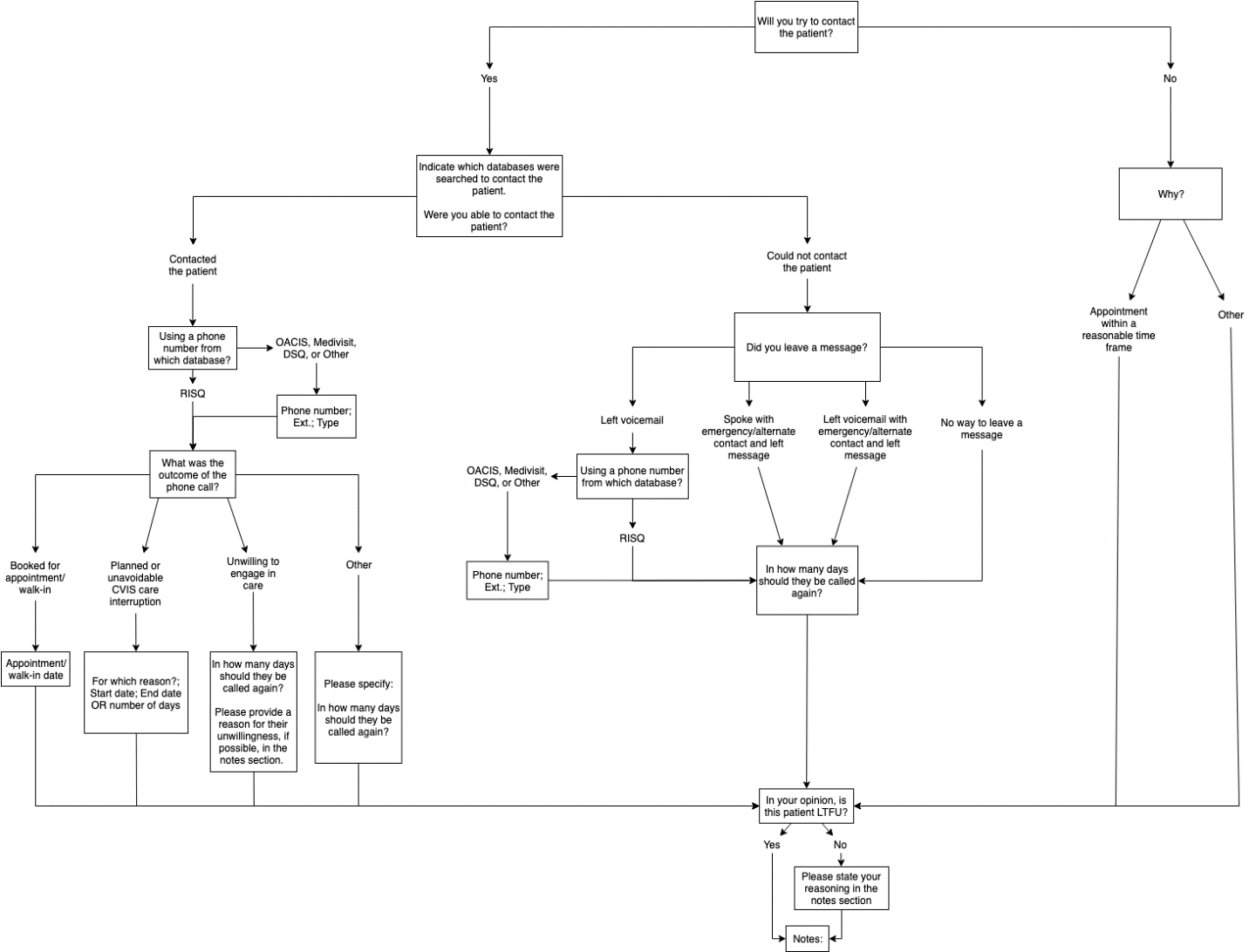
[v1.5](#v1_5)

###
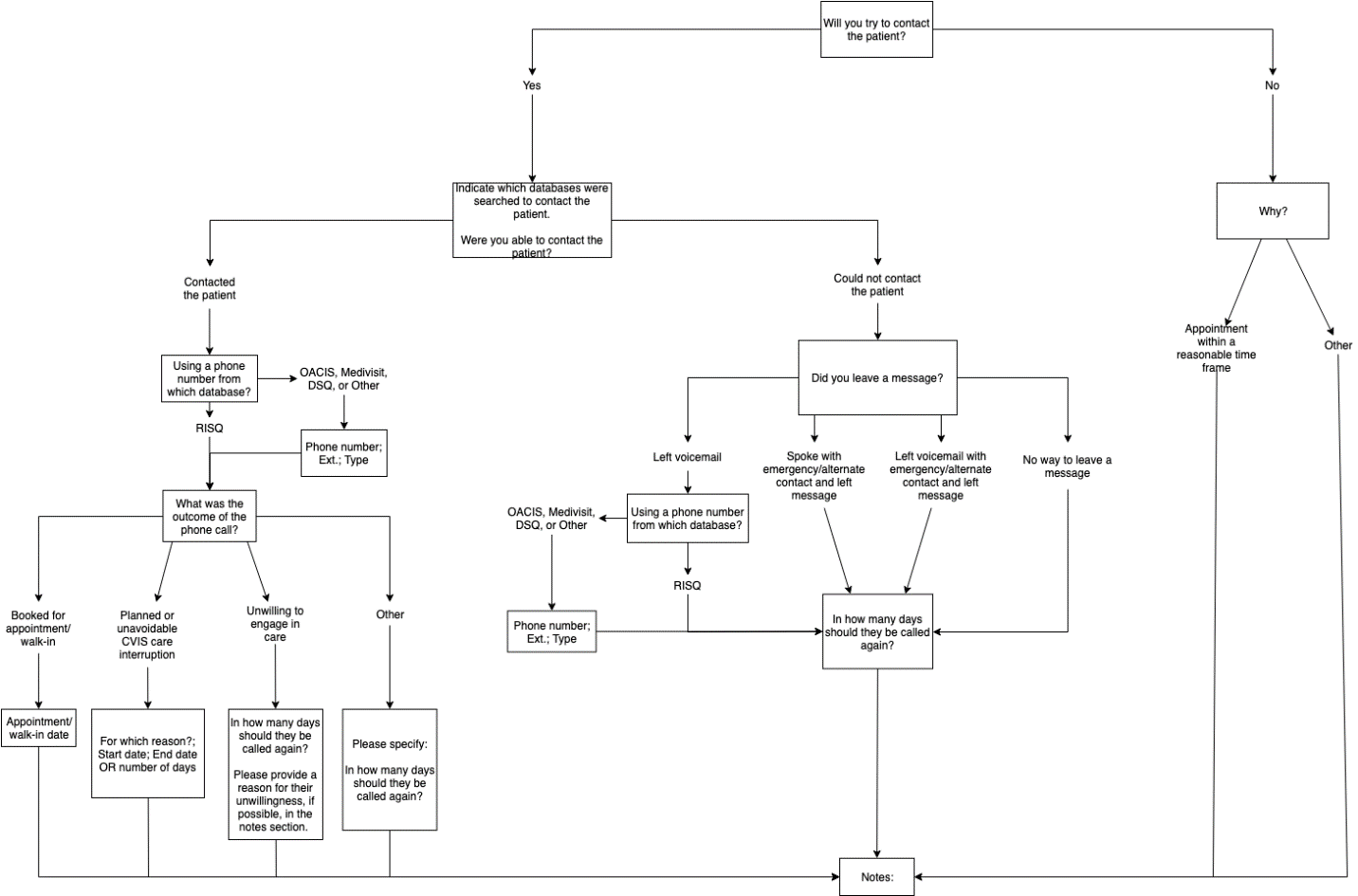
[v1.2](#V1_2)

###
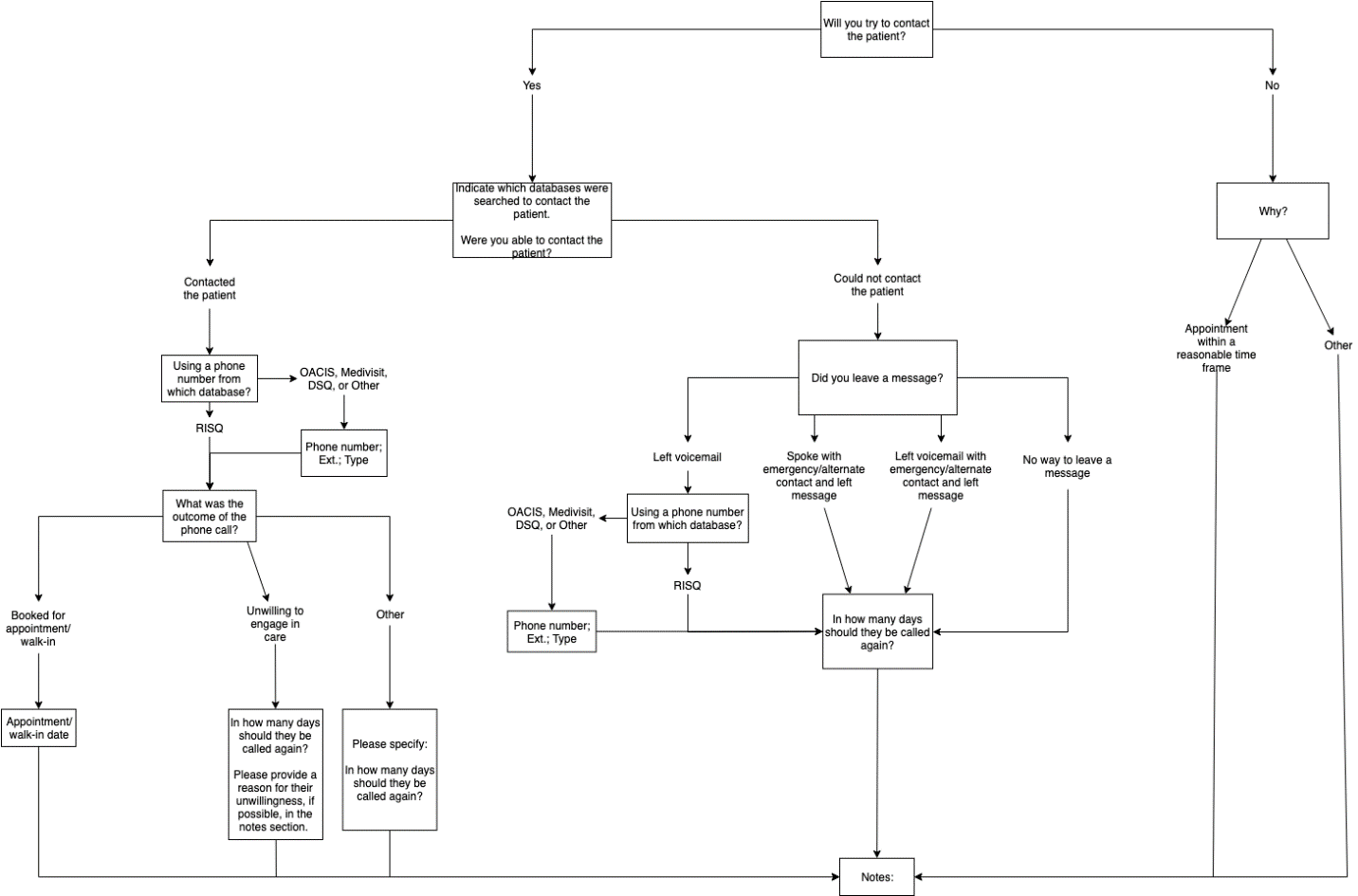
[v0.2](#v0_2)

###
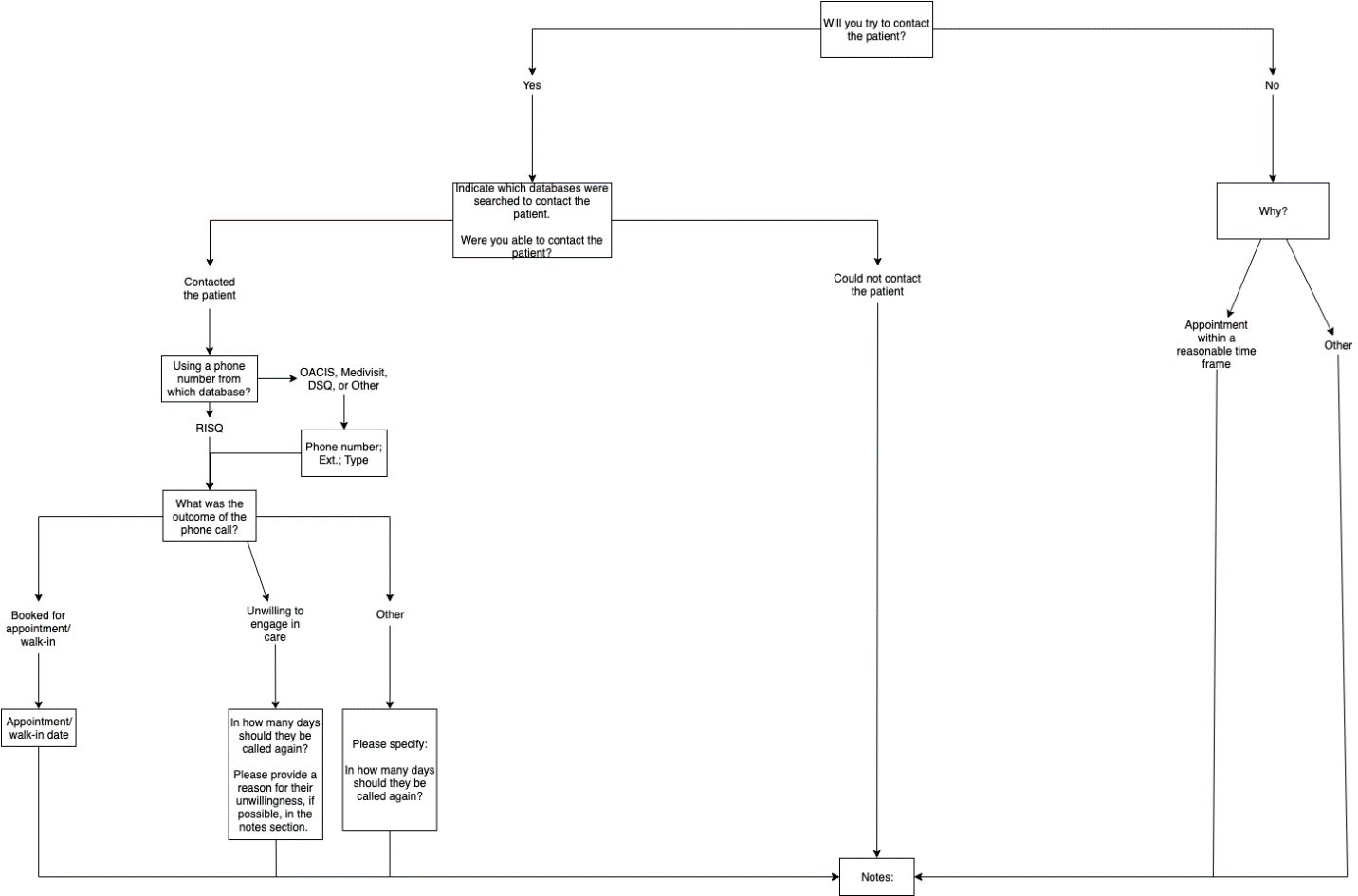
[v0.1](#v0_1)

###
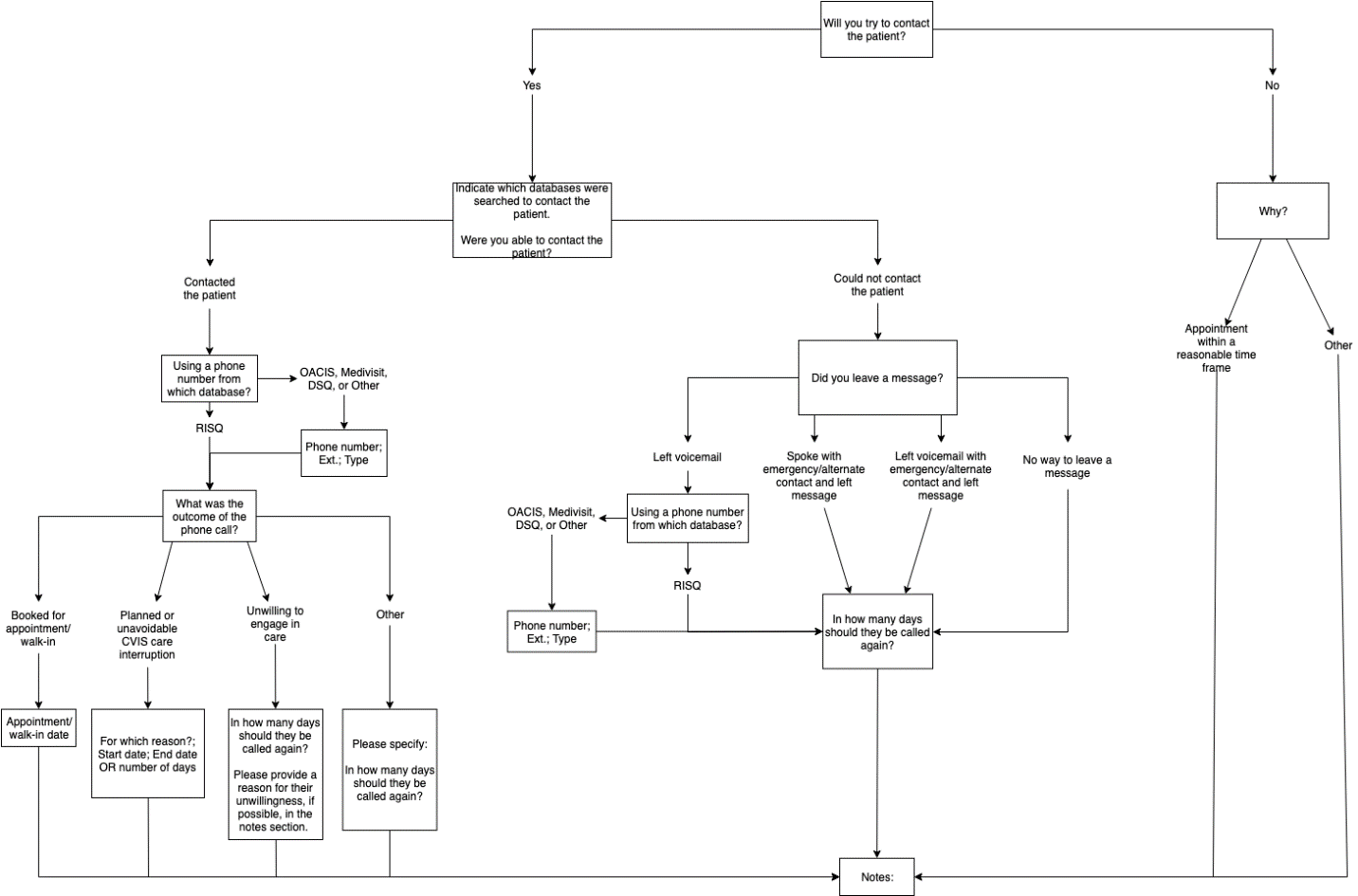
[**Initial request**](#InitialRequest1_0) **1.0 & 1.1**

## **Follow-up status**

### [v1.9](#v1_9)


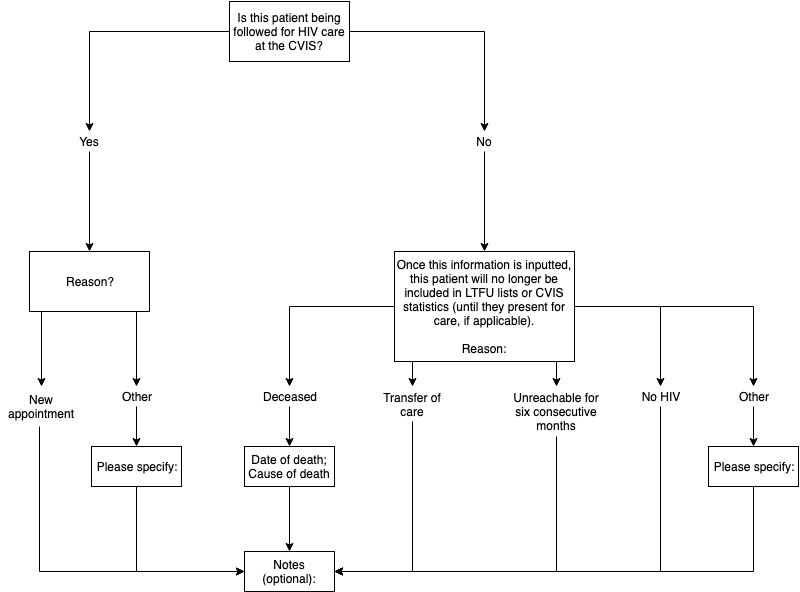


###
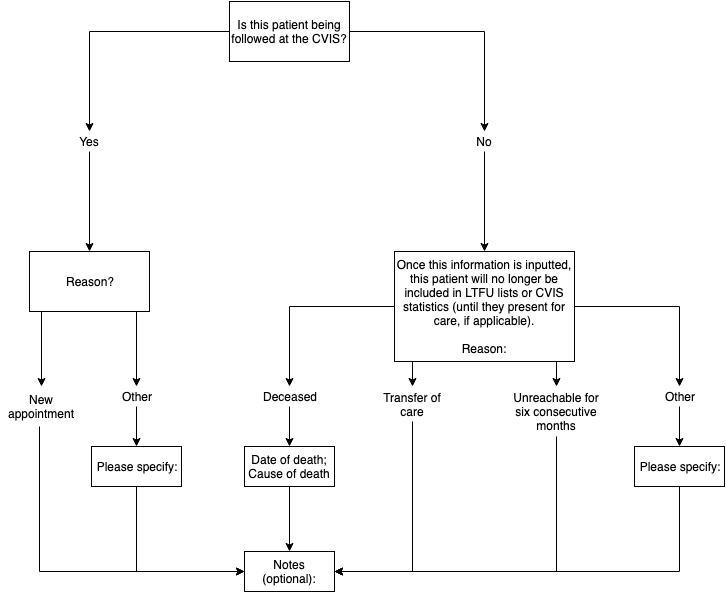
[**Initial request**](#InitialRequest1_0) **1.0 & 1.1**

# **Appendix 3: LTFU status bar functionality**

### **[Initial request 1.1](#InitialRequest1_1)**


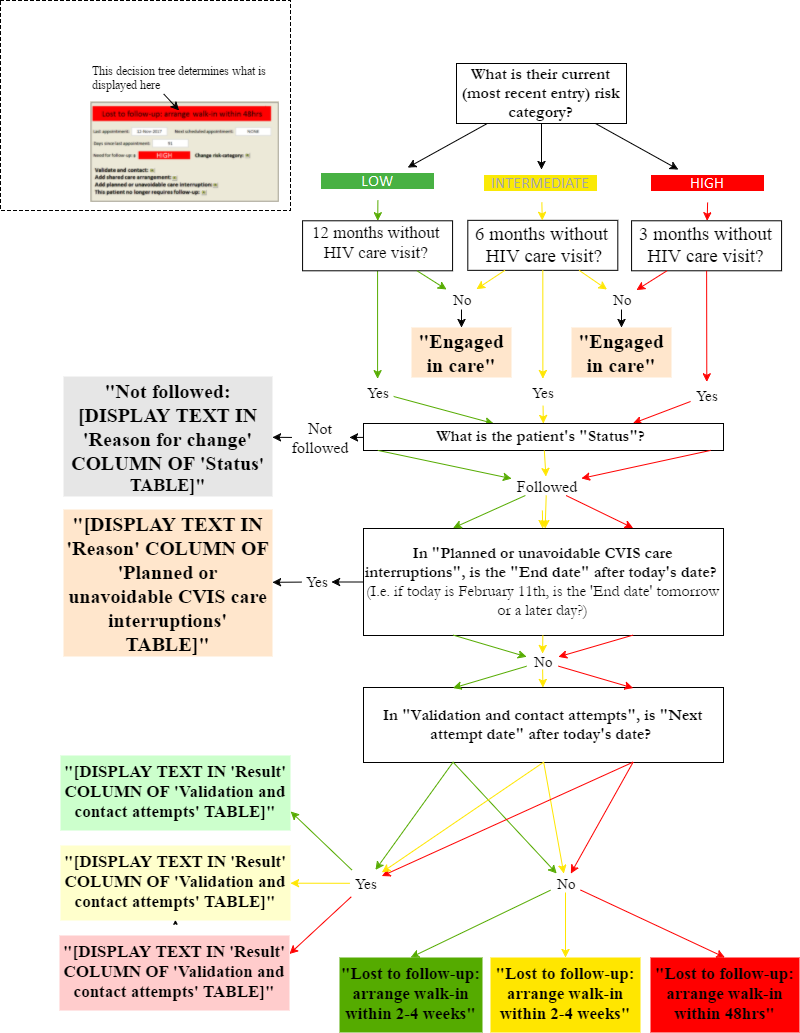


# **Appendix 4: Risk category heirarchy in RISQ**

## [v1.2 and later](#V1_2): Automatic risk category changes, after risk categories set either manually or automatically (not considering the UNKNOWN category)


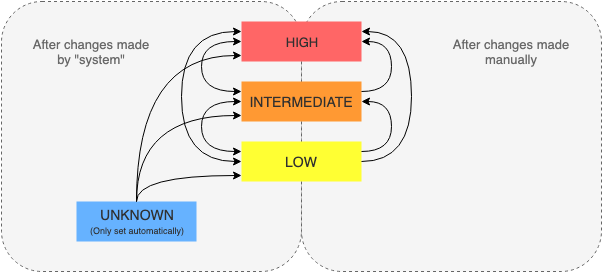


## [V0.2 to v1.1](#v0_2): Automatic risk category changes, after risk categories set either manually or automatically (not considering the UNKNOWN category)


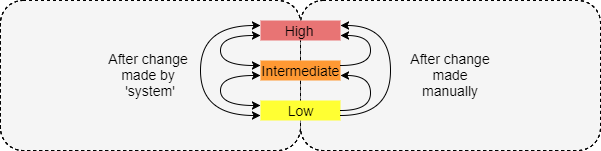


###
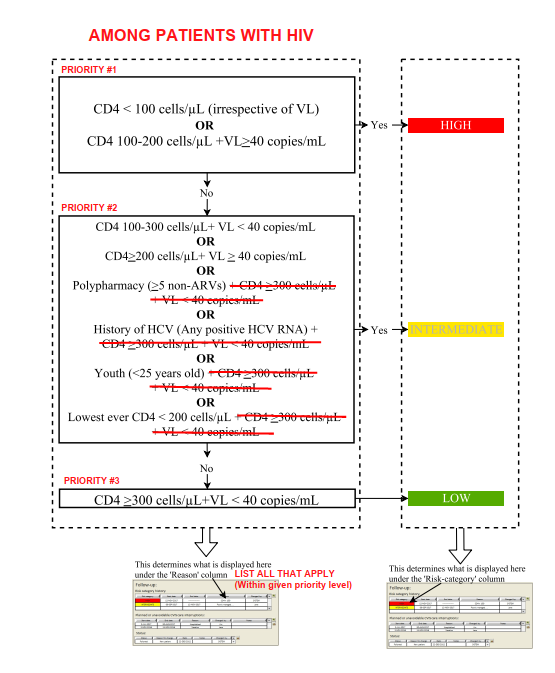
v1.1

## **[Initial request 1.1](#InitialRequest1_1)**


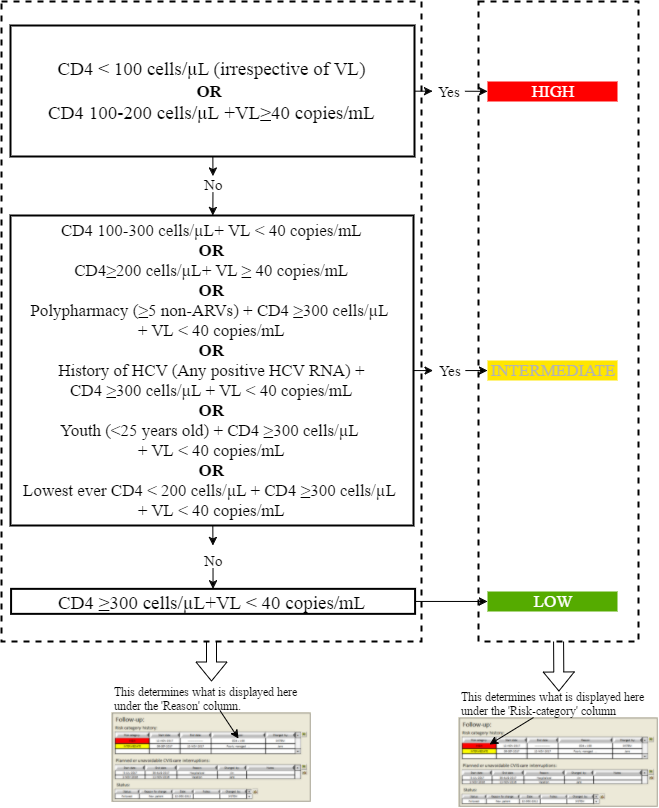


# **Appendix 5: Photos for specific errors**

## [v1.1](#V1_1)


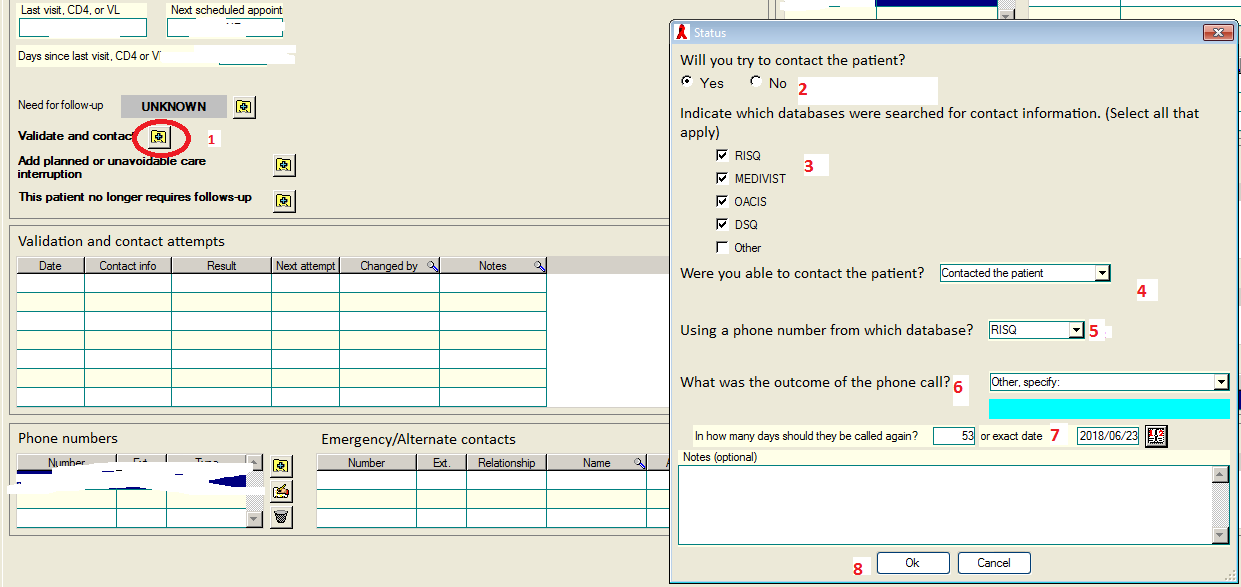


# **Appendix 6: Order of the LTFU list**

## [v1.0](#V1_0)

| **Order** | **Image/ Color** | **Status** | **Text in Instructions box** | **Category** | **Care interruption** | **Contact attempt** | **Last visit** | **CD4, VL** |
| --- | --- | --- | --- | --- | --- | --- | --- | --- |
| 100 |  | Followed | Lost to follow-up, arrange walk-in within 48hrs | High | no | no |  |  |
| 130 |  | Followed | Lost to follow-up, arrange walk-in within 2-4 weeks | Intermediate | no | no |  |  |
| 160 |  | Followed | Lost to follow-up, arrange walk-in within 4-6 weeks | Low | no | no |  |  |
| 200 |  | Followed | Contacted | High | no | Next Attempt Date : in the future |  |  |
| 230 |  | Followed | Contacted | Intermediate | no | Next Attempt Date : in the future |  |  |
| 260 |  | Followed | Contacted | Low | no | Next Attempt Date : in the future |  |  |
| 600 |  | Followed | Incarcerated | any except Unknown | Incarcerated until 2019 | no |  |  |
| 600 |  | Followed | Engaged in care | Any except Unknown | no | no | week ago |  |
| 700 |  | Followed | No visits | Any except Unknown | no | no | No visits |  |
| 700 |  | Followed | Category Unknown, no results for CD4 or VL | Unknown | any | any | any | No CD4s or VLs |
| 800 |  | Not followed | Not followed: [Reason] | any |  |  |  |  |
| 900 | Fleur | Not followed | Not followed: Deceased | any |  |  |  |  |
